# A novel effector, CsSp1, from *Bipolaris sorokiniana,* is essential for colonization in wheat and is also involved in triggering host immunity

**DOI:** 10.1111/mpp.13155

**Published:** 2021-11-06

**Authors:** Wanying Zhang, Haiyang Li, Limin Wang, Shunpei Xie, Yuan Zhang, Ruijiao Kang, Mengjuan Zhang, Panpan Zhang, Yonghui Li, Yanfeng Hu, Min Wang, Linlin Chen, Hongxia Yuan, Shengli Ding, Honglian Li

**Affiliations:** ^1^ Department of Plant Pathology, College of Plant Protection Henan Agricultural University/Collaborative Innovation Center of Henan Grain Crops/National Key Laboratory of Wheat and Maize Crop Science Zhengzhou China; ^2^ Department of Landscape Architecture and Food Engineering Xuchang Vocational Technical College Xuchang China; ^3^ Agriculture and Rural Affairs Bureau Xuchang China

**Keywords:** *Bipolaris sorokiniana*, CsSp1, effector, elicitor, plant immunity, salicylic acid, wheat root rot

## Abstract

The hemibiotrophic pathogen *Bipolaris sorokiniana* causes root rot, leaf blotching, and black embryos in wheat and barley worldwide, resulting in significant yield and quality reductions. However, the mechanism underlying the host–pathogen interactions between *B. sorokiniana* and wheat or barley remains unknown. The *B. sorokiniana* genome encodes a large number of uncharacterized putative effector proteins. In this study, we identified a putative secreted protein, CsSp1, with a classic N‐terminal signal peptide, that is induced during early infection. A split‐marker approach was used to knock out *CsSP1* in the Lankao 9‐3 strain. Compared with the wild type, the deletion mutant ∆*Cssp1* displayed less radial growth on potato dextrose agar plates and produced fewer spores, and complementary transformation completely restored the phenotype of the deletion mutant to that of the wild type. The pathogenicity of the deletion mutant in wheat was attenuated even though appressoria still penetrated the host. Additionally, the infectious hyphae in the deletion mutant became swollen and exhibited reduced growth in plant cells. The signal peptide of CsSp1 was functionally verified through a yeast YTK12 secretion system. Transient expression of CsSp1 in *Nicotiana benthamiana* inhibited lesion formation caused by *Phytophthora capsici*. Moreover, CsSp1 localized in the nucleus and cytoplasm of plant cells. In *B. sorokiniana*‐infected wheat leaves, the salicylic acid‐regulated genes *TaPAL*, *TaPR1*, and *TaPR2* were down‐regulated in the ∆*Cssp1* strain compared with the wild‐type strain under the same conditions. Therefore, CsSp1 is a virulence effector and is involved in triggering host immunity.

## Graphical abstract

CsSp1, a virulent effector in *Bipolaris sorokiniana*, also triggers immunity in plant cells.

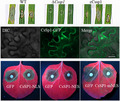

## 1 INTRODUCTION

*Bipolaris sorokiniana* (teleomorph: *Cochliobolus sativus*) is one of the main pathogens responsible for wheat root rot, crown rot, leaf spot, and black points of wheat, barley, and many other grass species (Acharya et al., 2011; Karov et al., 2009; Kumar et al., 2002, 2020; Yan et al., 2012). Diseases caused by *B. sorokiniana* have been shown to result in yield losses ranging from 10% to 20% under favourable conditions in Canada, the UK, Brazil, Mexico, Gambia, and southern Asia (Ghazvini & Tekauz, 2012; Kang et al., 2020; Karov et al., 2009; Murray et al., 1998; Sharma & Duveiller, 2010). On the northern plains of China, *B. sorokiniana* was determined to be the most abundant pathogen present in infected wheat roots and stems (Xu et al., 2018). *B. sorokiniana* accounts for 0.3%–66.7% of black point disease cases in China, based on screening of wheat cultivars and isolation of the pathogen from grains (Dai et al., 2011; Li et al., 2014; Luan et al., 2011; Zhang et al., 1990). In recent years, with changing climate conditions and returning straw to the field, wheat root rot has become more prevalent in different regions of China. In particular, in the Huanghuai wheat‐growing region of China, wheat root rot has become one of the major diseases (Guo, Yao, et al., 2019; Li et al., 2011; Wu et al., 2006; Zhang et al., 2007).

*B. sorokiniana* is a hemibiotrophic parasitic fungus, and its infection process is similar to that of *Magnaporthe oryzae* (Gupta et al., 2018). The conidia secrete mucus, adhere to the surface of host plants, and then germinate to form buds and germ tubes, which extend and produce multiple branches. Appressoria differentiate at the tops of branches, producing penetration pegs that directly penetrate the cuticle on the surface of host cells, and invasive mycelia extend inside or between host cells (Han et al., 2010; Kumar et al., 2002; Verma et al., 2020). It is rare for hyphae to infect through natural openings such as stomata. Due to heterokaryotic conditions, the morphology of isolates from the field widely vary. In a natural population of *B. sorokiniana*, the frequency of the greenish‐grey colony colour was 31.25%, followed by black (25%) and grey or white (18.75%), whereas brown was the least frequent colour (6.25%) (Verma et al., 2020).

It is well known that salicylic acid (SA) is involved in the plant immune response against biotrophic and hemibiotrophic pathogens, and is associated with the induction of plant systemic acquired resistance (SAR) (Alvarez, 2000; Meenakshi & Singh, 2013; Vlot et al., 2009). SA accumulates during both incompatible and compatible interactions between *B. sorokiniana* pathogens and host plants to facilitate resistance to spot blotches (Al‐Daoude, 2019; Al‐Daoude et al., 2018; Sahu et al., 2016). Lesion development is associated with the accumulation of host‐encoded pathogenesis‐related (PR) proteins and reactive oxygen species (ROS) (Ajith et al., 2003). Among the *PR* genes, *PR1*, *PR2*, and *PR5* are commonly used as markers for the activation of SAR (Zhang et al., 2010). On the other hand, pathogen effectors that suppress SA signalling without affecting SA biosynthesis are also known to exist (Kazan & Lyons, 2014). For example, when expressed in Arabidopsis, two effectors, *Hyaloperonospora arabidopsidis* effector HaRxL96 and *Phytophthora sojae* effector PsAvh163, suppress the pathogen‐mediated induction of marker genes such as *PR1* (Anderson et al., 2012). Many studies have shown that plant pathogens can manipulate SA signalling and that many effectors are involved in this process.

To overcome host detection and defence, most pathogens produce a range of secreted effectors and metabolites (Stergiopoulos & de Wit, 2009; Wit et al., 2010). Two kinds of effectors are found in the cytosol in host cells or the apoplastic space (Giraldo et al., 2013; Koeck et al., 2011; Zhang & Xu, 2014). A number of studies have revealed the function of intracellular effectors in filamentous plant pathogens (Bozkurt & Kamoun, 2020; Pramod et al., 2019). In oomycetes, the movement of effectors to host cells occurs via common amino acid sequence motifs, such as RxLR (Arg‐x‐Leu‐Arg), LxLFAK or the Crinkler motif (CRN), and ChxC (Jiang et al., 2008). In fungi, a small group of effectors from barley powdery mildew, wheat stem rust, and wheat leaf rust share a conserved motif, Y/F/WxC, that follows the secreted signal peptide (Godfrey et al., 2010). However, most fungal effectors lack conserved domains (Caillaud et al., 2012; Selin et al., 2016). In *M. oryzae*, effectors are delivered to the cytosol through a specific structure called the biotrophic interfacial complex (BIC) (Khang et al., 2010). Most of the effectors from pathogens contribute quantitatively to pathogen aggressiveness, but some of them, such as Cmu1 and Scc1 in *Ustilago maydis* (Djamei et al., 2011; Redkar et al., 2015), BAS107 in *M. oryzae* (Giraldo et al., 2013), MiSSP7 in *Laccaria bicolor*, which functions as a negative regulator of jasmonic acid (JA)‐induced gene regulation in the nucleus (Plett et al., 2011), PcCRN4 in *Phytophthora capsici*, which suppresses host defence and induces cell death in the plant nucleus (Mafurah et al., 2015), SsSSVP1 in *Sclerotinia sclerotiorum* (Lyu et al., 2016), VdSCP7 in *Verticillium dahliae* (Zhang et al., 2017), and SCRE1 in *Ustilaginoidea virens*, which inhibits host immunity and suppresses the immunity‐associated hypersensitive response (HR) via the plant nucleus (Zhang, Yang, et al., 2020), have been identified as potential nuclear‐localized regulators of the host cell targeting process (Diaz‐Granados et al., 2020). The conserved targeting mechanism of a common host protein network for convergent effectors from the eubacteria *Pseudomonas syringae*, the oomycete *Hyaloperonospora arabidopsidis*, and the ascomycete *Golovinomyces orontii* has been explored in the model plant species *Arabidopsis thaliana* (Weßling et al., 2014). The wheat blue dwarf phytoplasma effector SWP11 induces three *PR* genes, *PR1*, *PR2*, and *PR3*, to trigger plant immunity (Wang et al., 2018). In general, effectors suppress or induce plant cell death mostly through manipulation of the host immune system (Knig et al., 2020; Sharpee & Dean, 2016; Shen et al., 2018; Wang et al., 2011). Some effectors are recognized by the plant immune system through specific resistance proteins and are termed avirulence proteins (Boller & Felix, 2009; Malik et al., 2020). *AvrPrm3* in *Blumeria graminis* is recognized by the *Pm3* resistance gene in wheat (Bourras et al., 2015), while *Avr2* and *Avr3* in *Fusarium oxysporum* f. sp. *lycopersici* interact with *I‐2* and *I‐3* resistance genes in tomato, respectively (Houterman et al., 2008, 2010; Rep et al., 2005).

There are few reports on the mechanism underlying the molecular regulation of the pathogenicity of *B. sorokiniana* in wheat root rot. A comparative analysis of candidate effector‐coding genes in the genomes of five *Bipolaris* species revealed 289 putative small‐molecular‐weight secreted proteins in *B. sorokiniana*, 167 of which were unique. There were significantly more secreted proteins than other pathogens of the same genus, and the functions of these secreted proteins have not been reported (Condon et al., 2013). Pathak et al. (2020) investigated the secretome of 196 proteins predicted to be present in *B. sorokiniana* in silico. The *ToxA* gene encodes a host‐selective toxin (HST) that functions as an effector, and *B. sorokiniana* has been shown to carry this gene (Sudhir et al., 2020). However, no experimental evidence has been shown for secreted proteins in *B. sorokiniana*. In this study, we identified a gene, *CsSP1*, encoding a small‐molecular‐weight secreted protein in *B. sorokiniana* that was highly expressed in the infection stage. This protein is involved in pathogenicity and acts as a novel elicitor triggering the host immune system, and this protein might be a candidate for the control of plant disease.

## 2 RESULTS

### 2.1 *CsSp1* is highly expressed during infection

**FIGURE 1:**
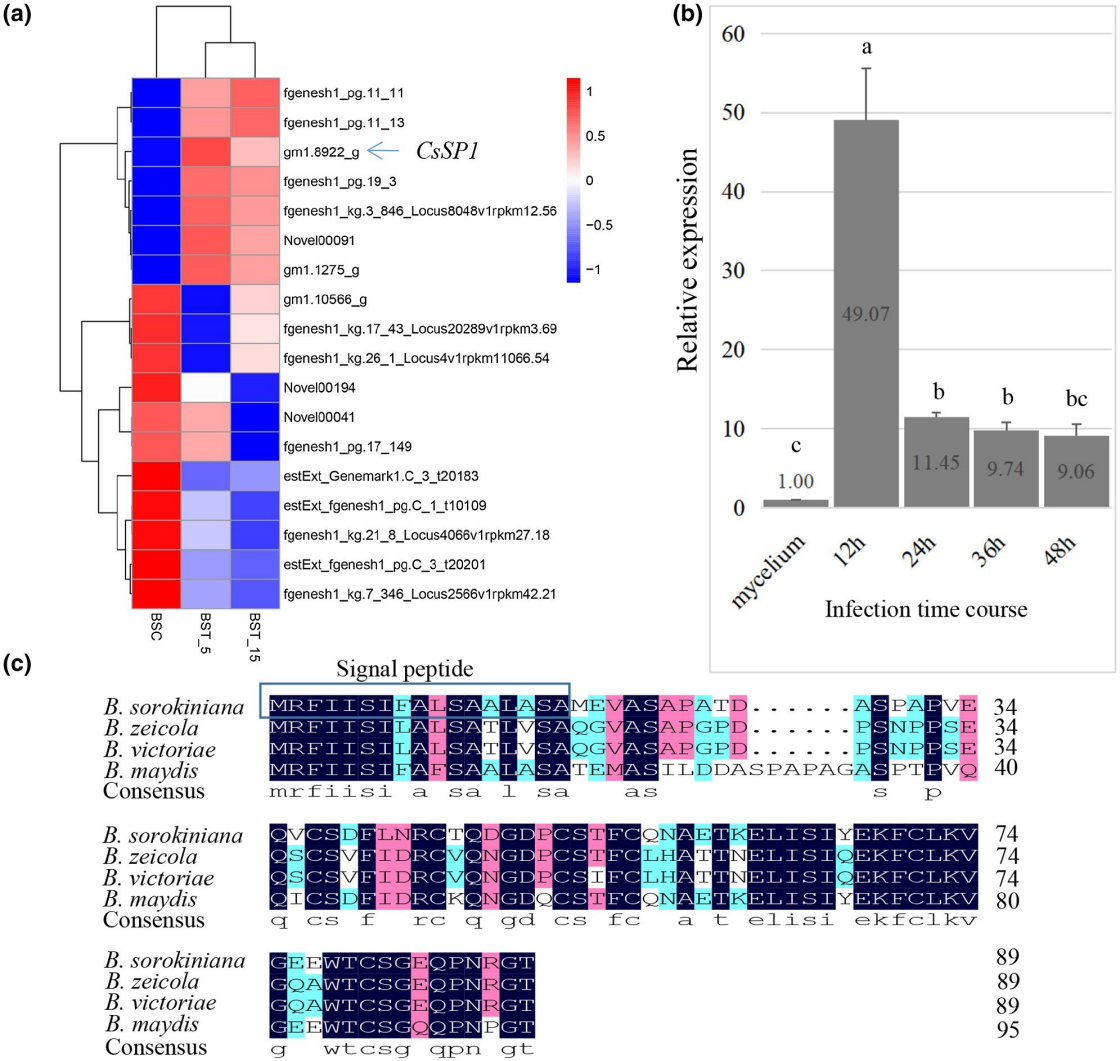
Differentially expressed genes (DEGs) in the transcriptome and *CsSP1* feature analysis. (a) Cluster analysis of DEGs from the wheat Aikang 58 cultivar infected with *Bipolaris sorokiniana*. (b) Reverse transcription quantitative PCR (RT‐qPCR) confirmation of the expression pattern of *CsSP1* in planta. Aikang 58 leaves were inoculated with 10^5^ spores/ml of *B. sorokiniana* Lankao 9‐3. The inoculated leaves were placed in a moist chamber in the dark for 24 h and then kept in a greenhouse at 25°C (47% humidity) with a 16 h light/8 h dark photoperiod. The leaves were sampled at 12, 24, 36, and 48 h for total RNA extraction, while mycelia cultured for 2 days in YEPD were used for fungal RNA extraction. The experiments were repeated three times. The expression levels were calculated using the 2^−∆∆^ *^C^* ^t^. Significant differences calculated by Tukey's LSD, *p* < 0.05. (c) Alignment of CsSp1 orthologues from *Bipolaris* spp. was performed using DNAMAN. BSC: *B*. *sorokiniana* mycelia from liquid medium served as controls. BST_5 and BST_15: assays of Aikang 58 root and stem base samples from plants growing in pots at 5 and 15 days after *B. sorokiniana* infection. Signal peptides were predicted by SignalP v. 4.0 software (http://www.cbs.dtu.dk/services/SignalP/)

To evaluate gene expression during interactions with wheat, RNA sequencing (RNA‐Seq)‐based transcriptome analysis was performed using roots and basal stems of Aikang 58 wheat seedlings grown in pots at 5 days and 15 days after soil inoculation with *B. sorokiniana* (Figure S1a–d). Analysis of transcriptome data revealed very few *B. sorokiniana* sequence reads. A gene encoding a small protein (9.7 kDa) with a predicted N‐terminal signal peptide was highly expressed during the *B. sorokiniana* infection stage (Figure 1a). We designated this protein as *B. sorokiniana*‐secreted protein 1 (CsSp1). To verify the expression pattern of *CsSp1* during wheat leaf infection, reverse transcription quantitative (RT‐qPCR) was conducted. The results showed that the expression level of *CsSP1* increased more than 40‐fold at 12 h postinoculation (hpi), decreased at 48 hpi and remained higher than that of hyphae grown in vitro (Figure 1b). BLASTP analysis of the CsSp1 protein indicated that the amino acid identity ranged from 71.58% to 73.03% with that from *Bipolaris maydis*, *Bipolaris victoriae*, and *Bipolaris zeicola*. Amino acid sequence alignment was performed via DNAMAN (Figure 1c), and a phylogenetic tree was constructed based on the amino acid alignment (Figure S2c). The results showed that CsSp1 is a protein specific to plant‐pathogenic fungi and is found only in the *Bipolaris* genus.

### 2.2 Generation of *CsSP1* deletion mutants and functional complementation of ∆*Cssp1*

**FIGURE 2:**
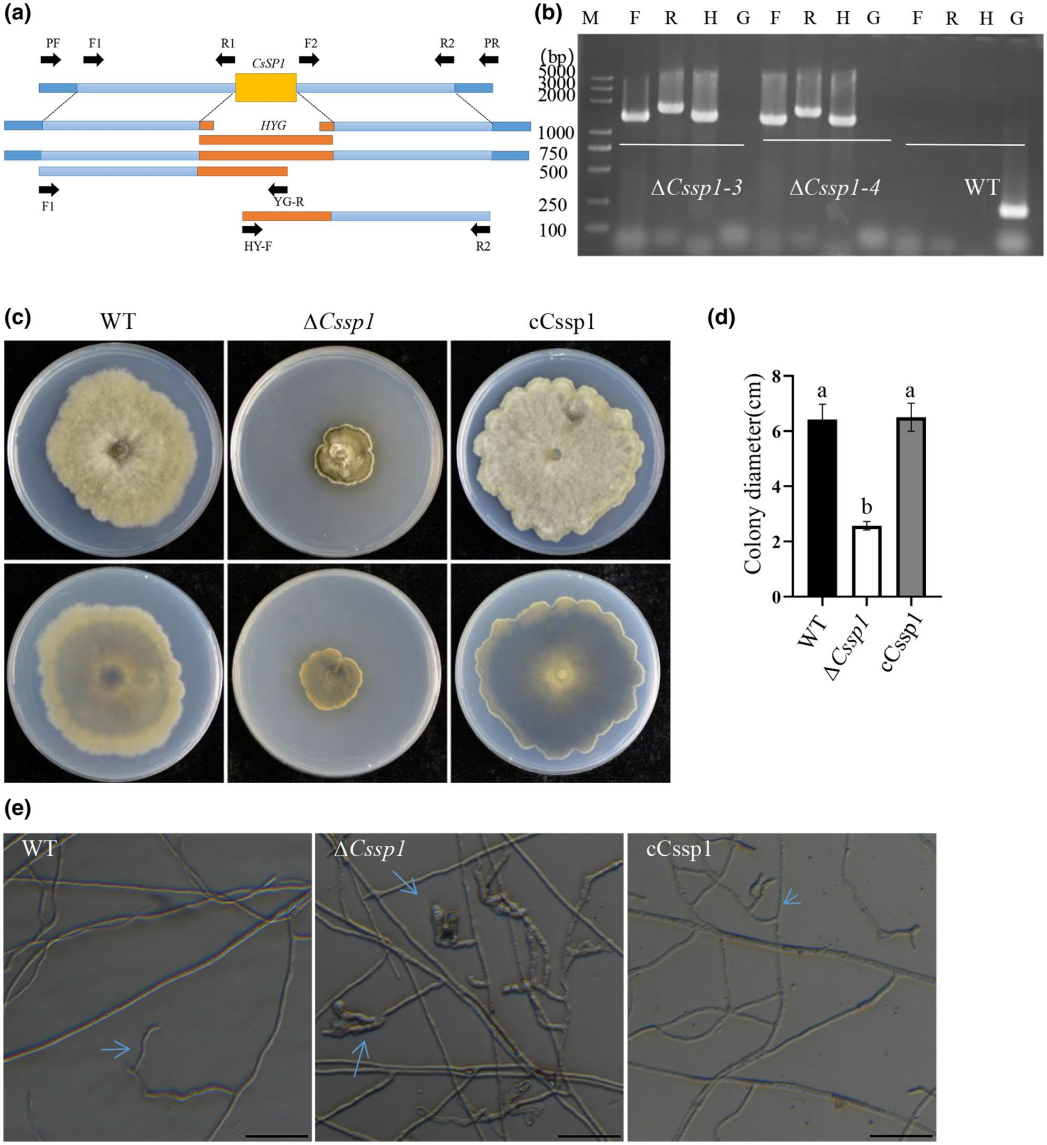
*CsSP1* knockout and characterization. (a) Diagram of the gene deletion strategy for *CsSP1*. The primers used for gene replacement and mutant screening are indicated by the arrows and are listed in Table 2. (b) Agarose gel electrophoresis of PCR products from genomic DNA templates. Lane H, primers HYG‐F/HYG‐R for hygromycin resistance gene; lane F and lane R, CsSP1‐PF/H855R and H856F/CsSP1‐PR, respectively, for positive screening; lane G, CsSP1‐NF/CsSP1‐NR for negative screening. ∆*Cssp1‐3* and ∆*Cssp1‐4* are two candidates: wild‐type (WT) strain Lankao 9‐7; M, molecular markers; H, hygromycin resistance gene; F, upstream; R, downstream; G, *CsSP1* gene. (c, d) The colony morphology and growth rate calculated for 90 mm potato dextrose agar (PDA) plates after 7 days. The bars indicate the standard errors. The experiments were repeated three times. Significant differences calculated by Tukey's LSD, *p* < 0.05. (e) Morphology of hyphal tips on PDA plate. Bars, 50 μm

To characterize the biological function of *CsSP1*, a split‐marker approach was applied to knock out *CsSP1* in the wild‐type (WT) strain Lankao 9‐3 (Figure 2a). Through polyethylene glycol (PEG)‐mediated protoplast transformation, we successfully obtained *CsSP1* deletion mutants ∆*Cssp1‐3* and ∆*Cssp1‐4*, in which the *CsSP1* gene had been successfully replaced with the hygromycin gene cassette (Figure 2b). Compared with the WT strain, the deletion strain ∆*Cssp1* grew more slowly on potato dextrose agar (PDA) (Figure 2c,d). Compared with that of the WT hyphae, the tips of the ∆*Cssp1* hyphae were enlarged on the PDA plates (Figure 2e). To demonstrate that these changes were caused by the deletion of the target gene *CsSP1*, we generated a complementary expression construct. The *CsSP1* open reading frame (ORF) with the 1.8 kb promoter and without a stop codon was amplified via PCR from the WT strain, cloned, and ligated into a pYIP‐102 expression vector via fusion to a FLAG/S tag. The resulting construct was sequenced and transformed into the ∆*Cssp1‐4* deletion mutant to create cCssp1. The growth phenotypes of the positive complementary transformants with G418 resistance were identical to those of the WT (Figure 2c–e). Therefore, *CsSP1* is an essential growth‐related gene.

### 2.3 CsSp1 is involved in *B. sorokiniana* conidial regulation

**FIGURE 3:**
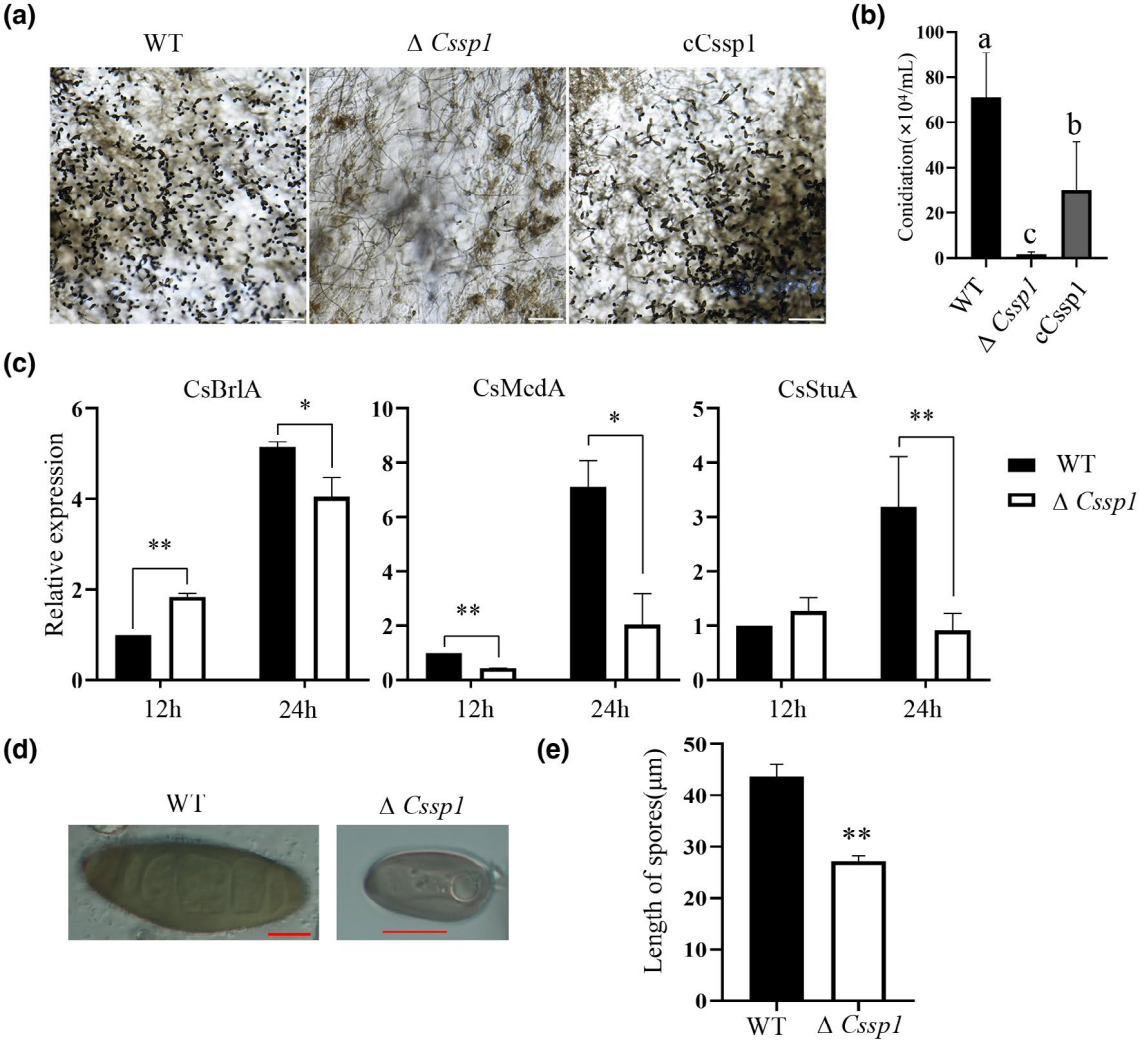
∆*Cssp1* spore morphology, conidiation, and potential regulation in the wild type (WT) and complemented (cCssp1) strains. (a) Conidial production was examined via microscopy. Bars, 200 μm. (b) The number of conidia were calculated. Significant differences calculated by Tukey's LSD, *p* < 0.05. (c) Reverse transcription quantitative PCR (RT‐qPCR)‐based measurement of the expression of genes involved in the regulation of sporulation from the total RNA extracted from mycelia. (d) Spore morphology. Bars, 10 μm. (e) Length of spores. The experiment was repeated three times, and 100 spores were counted each time. **p < 0.01, *p < 0.05 (*t* test)

To determine other biological functions of CsSp1, we counted the number of spores present on PDA plates. Compared with the WT, ∆*Cssp1* exhibited less sporulation (Figure 3a,b) and, in terms of morphology, the spores were smaller than those of the WT (Figure 3d). To explain the decrease in spore production, we measured the expression levels of the orthologous genes *CsBrlA*, *CsMedA*, and *CsStuA* (Wang et al., 2015), which are essential for the positive regulation of sporulation in both mutant and WT *B. sorokiniana*. The results showed that the expression levels of these candidate genes significantly decreased after 24 h of cultivation (Figure 3c). Taken together, these results confirmed the previous results in which CsSp1 is involved in the regulation of spore formation in *B. sorokiniana*.

### 2.4 CsSp1 is a virulence factor needed for full virulence of *B. sorokiniana*

**FIGURE 4:**
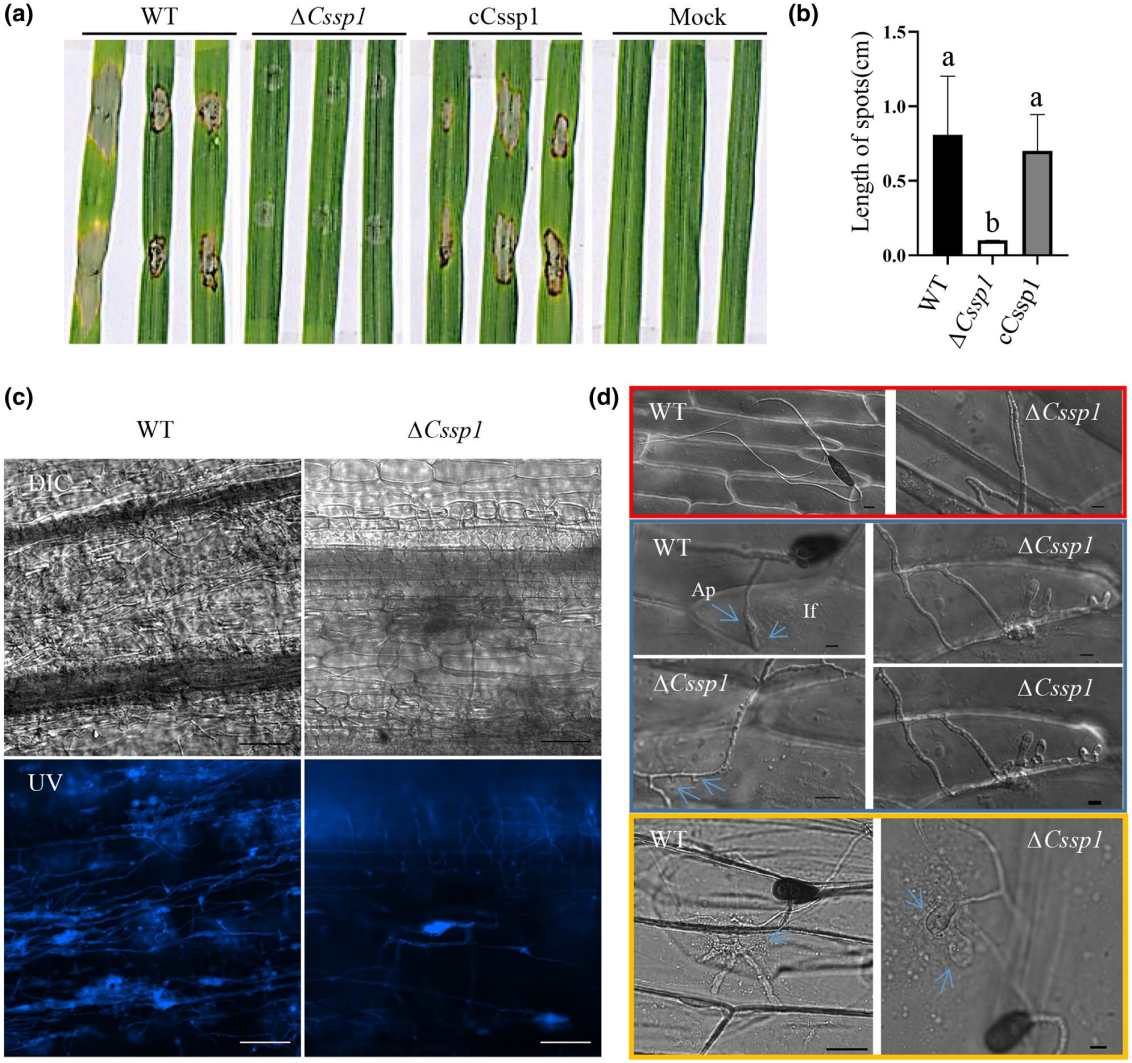
Pathogenicity tests. (a) Detached leaves from Aikang 58 seedlings in a tray were inoculated with drops of a spore suspension (3 × 10^4^/ml) of wild type (WT), deletion mutant Δ*Cssp1*, complemented strain cCssp1, or double deionized water as a mock control. The tray was kept in a moist chamber under darkness for 1 day and then moved to a greenhouse under a 16 h light/8 h dark photoperiod for 2 days. (b) Lesion lengths on wheat leaves were measured at 3 days postinoculation. Different letters indicate significant differences calculated by Tukey's LSD, *p* < 0.05. The experiments were repeated three times. (c) Mycelial expansion in barley leaves at 24 h after inoculation with fungal agar blocks. Fluorescent 7‐GFE staining was performed as described in Experimental Procedures. Bars, 100 μm. (d) Inner epidermis of onion bulbs 24 h after being inoculated with drops of spore suspensions (5 × 10^4^ cells/ml). Ap, appresorium; If, invasive hypha. Bars, 20 μm

To evaluate the role of ∆*Cssp1* in pathogenesis, we subjected wheat seedlings to a soil inoculation assay involving the application of 5‐mm diameter fungal agar plugs to the stem bases. The pathogenicity test indicated that the virulence to wheat rot of ∆*Cssp1* was nearly completely lost (Figure S3a). The leaves were inoculated with spore suspensions (3 × 10^4^ spores/ml), and the results showed that the ∆*Cssp1* mutants caused only tiny black spots on the leaves, while the WT produced larger spots (Figure 4a,b; Figure S3b). To observe the details of the hyphae, we inoculated barley leaves with a fungal agar block, and the infection progress was monitored and recorded (Figure 4c). After decolourization, the inoculated leaves were stained with solophenyl flavine 7GFE fluorescent dye (Figure 4c). Limited extension of invasive hyphae in the leaves was observed in the ∆*Cssp1* mutant compared with the WT, and infectious hyphae extended throughout the barley leaves. The infectious hyphal extension of ∆*Cssp1* was slower than that of the WT. To further characterize infection events of the *CsSP1* deletion mutant, the inner epidermis of onion bulbs was inoculated with a spore suspension (Figure 4d). The spores germinated normally; however, the morphology of the hyphal tips was altered, and the tips changed direction. ∆*Cssp1* displayed abnormal curving or swelling that seemed to initiate appressorium differentiation that had failed. The proportion of normal appressoria in ∆*Cssp1* was significantly lower than that in the WT (Figure S3d). Compared with the WT hyphae, the invasive ∆*Cssp1* hyphae were swollen and stunted, and extended more slowly (Figure 4h). Therefore, the *CsSP1* gene encodes a virulence factor involved in vegetative development and infection structure.

### 2.5 CsSp1 is a secreted effector that localizes to the nucleus and cytoplasm of host cells

**FIGURE 5:**
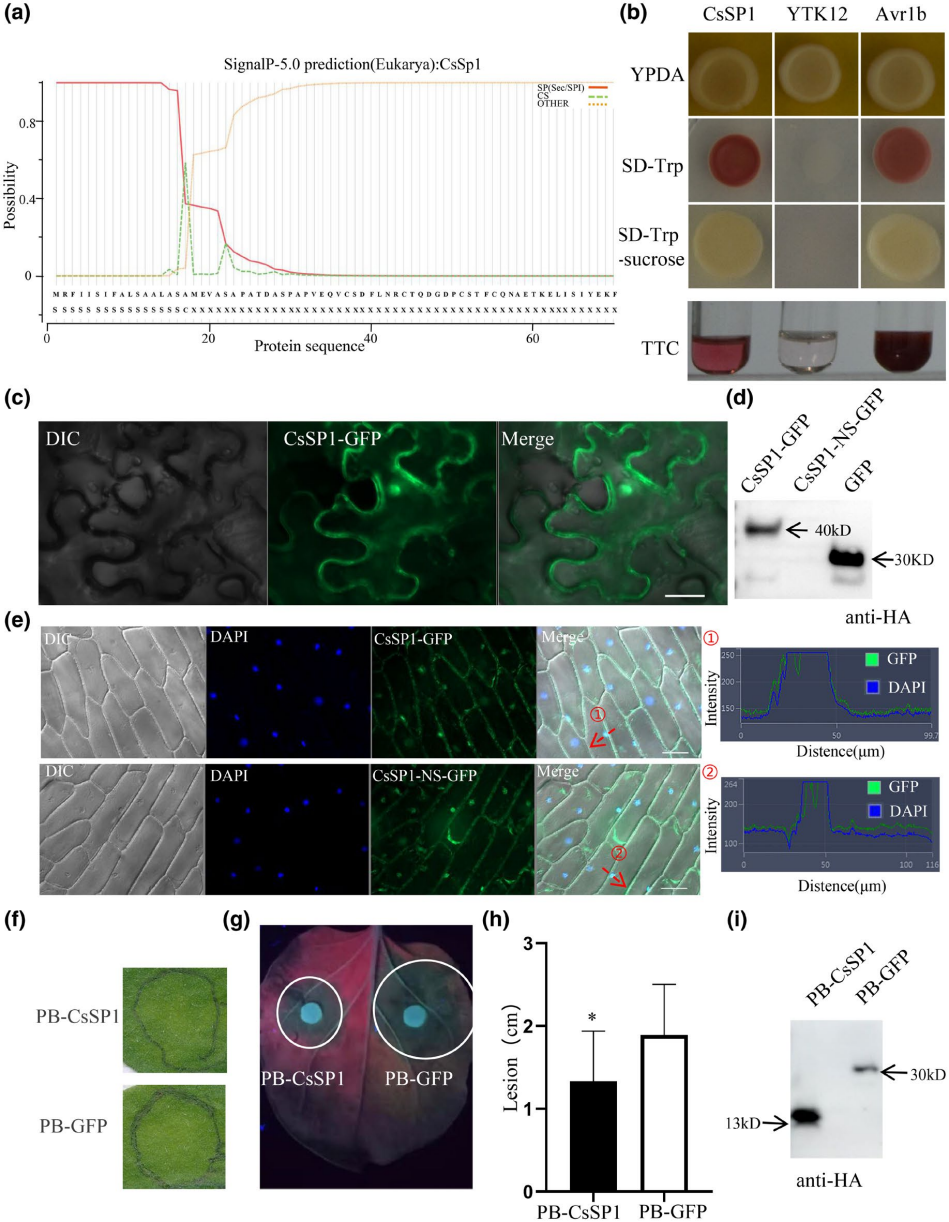
Functional identification of CsSp1 secretion, translocation, and elicitor characteristics. (a) The signal peptide of CsSp1 between 17 and 18 amino acid residues was predicted by SignalP. (b) Validation of signal peptides in a yeast system. If SUC2 invertase is secreted extracellularly in yeast, TTC can be reduced to red TTF. CsSp1, CsSp1 signal peptide with the expression vector pSUC2‐CsSP1‐SP in yeast; YTK12, empty yeast; Avr1b, avirulence gene b signal peptide, which served as a positive control. (c) Subcellular localization of CsSP1‐GFP in *Nicotiana benthamiana*. (d) Western blot analysis confirming protein expression with the PVX‐HA vector with an HA tag using protein from *N. benthamiana* leaves 48 h after *Agrobacterium tumefaciens* injection and leaves treated at 95°C for 5 min before protein extraction. CsSP1‐GFP and PVX‐CsSP1‐GFP include signal peptides. Mouse anti‐HA antibodies (M20003) and goat anti‐mouse secondary antibodies (IgG horseradish peroxidase conjugate; M21001) with working concentration of 1:5000 from Abmart were used (http://www.ab‐mart.com/). CsSP1‐NS, PVX‐CsSP1‐NS‐GFP lacking a signal peptide. GFP, PVX‐GFP. (e) Subcellular localization of CsSP1‐GFP in onion epidermis cells. The constructs used were the same as those in (d). The onion epidermis was treated with the corresponding *Agrobacterium*. Mock, MMA buffer treatment. Bars, 50 μm. (f) Characteristics of *N. benthamiana* at 6 days after being injected with *A. tumefaciens*. PB‐CsSP1 did not elicit a hypersensitive response (HR). PB‐GFP was used as a control. (g) *Phytophthora capsici* infection of *N. benthamiana* leaves injected with *A. tumefaciens*. (h) Diseased lesion areas were measured and calculated for the CsSp1 and GFP controls. *p < 0.05 (*t* test). The experiments were repeated three times. (i) PB‐HA detected via *N. benthamiana* anti‐HA antibodies from leaves 48 h after injection

Further bioinformatics analysis indicated that the protein encoded by *CsSP1* was smaller than 10 kDa and lacked similarity to proteins of known function. The protein has a 17 amino acid N‐terminal signal peptide sequence (Figure 5a), six predicted O‐glycosylation sites, and one serine phosphorylation site (Figure S5a,b and Table 1).

**TABLE 1:** The predictions for O‐glycosylation sites

Seq name	Residue	GlcNAc	Potential
N16	S	+	0.4481
N22	S	+	0.4431
N26	T	+++	0.5447
N29	S	++	0.4863
N45	T	+	0.3650
N89	T	+++	0.4250

To verify the function of the predicted signal peptide of *CsSP1*, the DNA fragment encoding the signal peptide was introduced into a pSUC2 vector (yielding a pSUC2‐CsSP1‐SP construct), which was subsequently transformed into yeast strain YTK12 to examine secreted invertase activity. Avr1b‐SP was used as a positive control. Growth tests on synthetic tryptophan (Trp) dropout agar medium plates showed that CsSP1‐SP and Avr1b‐SP restored the secretion of invertase and resulted in yeast growth on sucrose medium (Figure 5b). The activity of secreted invertase was also measured by the reduction of 2,3,5‐triphenyltetrazolium chloride (TTC), and the secreted invertase of the transformant containing Avr1b‐SP and CsSP1‐SP was measured by TTC assays (Figure 5b). CsSP1‐SP restored invertase such that TTC became red insoluble triphenylformazan (TTF) (Figure 5b). Thus, the CsSp1 signal peptide is functional.

To determine the subcellular localization of CsSp1, a C‐terminal green fluorescent protein (GFP) fused to CsSP1 was used. *CsSP1* was cloned and ligated into a PVX‐GFP‐3HA plant expression vector, which was then transiently expressed through agro‐infiltration in *Nicotiana benthamiana* leaves (Figure 5c). The signals from recombinant CsSp1‐GFP in *N. benthamiana* accumulated strongly in the nucleus with a cytosolic background (Figures 5c and S4a). Similarly, we constructed PVX‐CsSP1‐NS‐GFP (CsSp1 with no signal peptide, with GFP at fused at the C‐terminus) and PVX‐GFP expression vectors. In contrast to CsSp1‐GFP, PVX‐CsSP1‐NS‐GFP showed no fluorescence. Western blot analysis with anti‐HA antibodies confirmed the expression of these proteins in vivo, with the exception of PVX‐CsSP1‐NS‐GFP (Figure 5d). Similarly, the inner epidermis of onions was infected through agro‐infiltration to observe the subcellular localization of the gene product, and the results showed that CsSp1 was located in the nucleus and cytoplasm of onion cells (Figure 5e).

### 2.6 In planta expression of *CsSp1* suppresses pathogen extension

**FIGURE 6:**
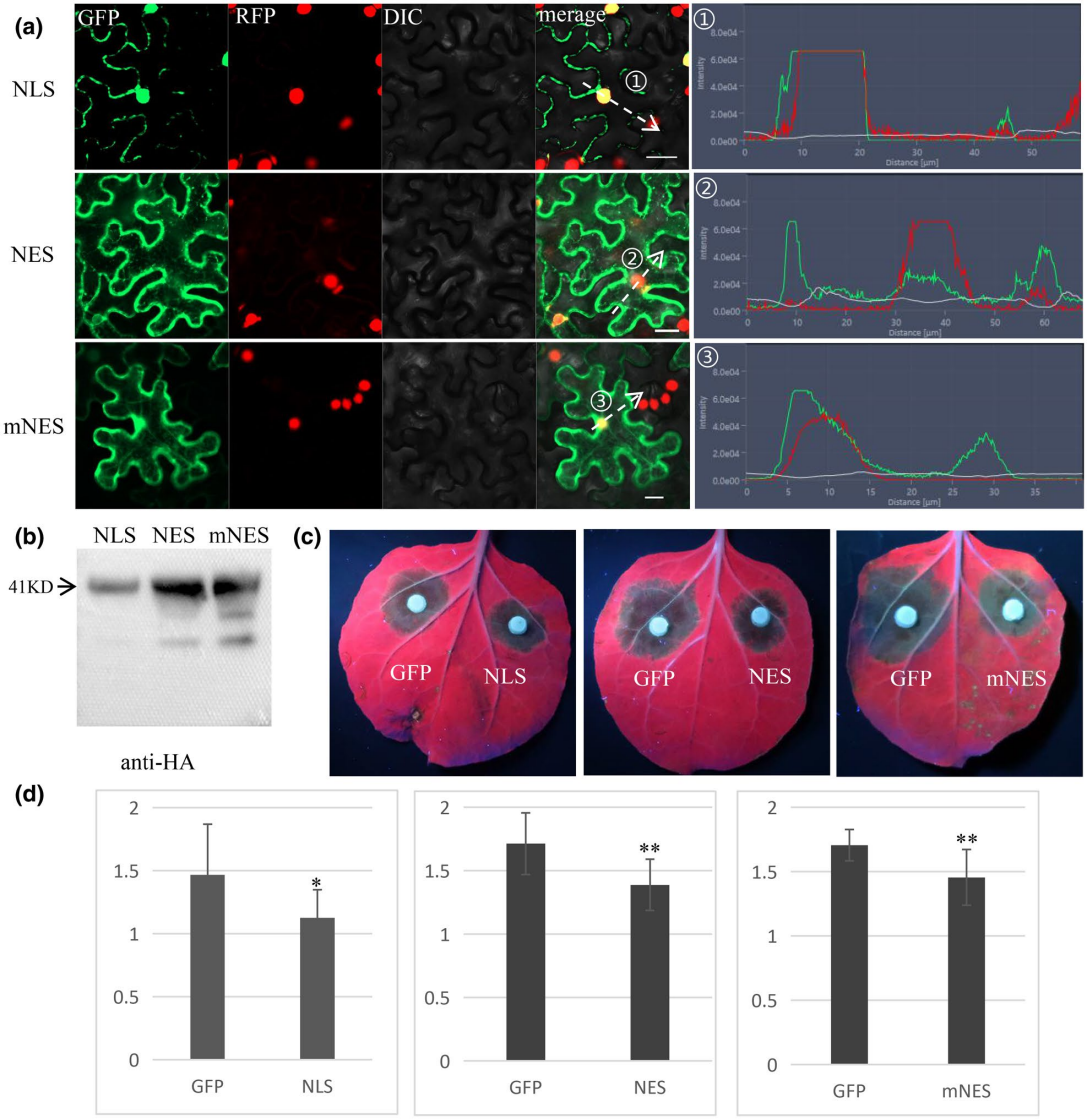
(a) Subcellular localization of CsSp1 mutants in *Nicotiana benthamiana*. NLS, CsSP1‐NLS‐GFP. NES, CsSP1‐NES‐GFP. mNES, CsSP1‐NES‐GFP. Bars, 20 μm. (b) Western blot analysis confirming protein expression with an anti‐HA tag antibody using protein from *N. benthamiana* leaves. (c) *Phytophthora capsici* infection of *N. benthamiana* leaves injected with *Agrobacterium tumefaciens*. (d) Diseased lesion areas were measured and calculated for *CsSp1* mutants and the GFP controls. **p < 0.01, *p < 0.05 (*t* test). The experiments were repeated three times

*CsSP1* was amplified via PCR and cloned into a vector PB‐3HA plant expression. Agro‐infiltration of PB‐CsSP1 did not induce cell death (Figure 5f). Because *B. sorokiniana* itself does not infect *N. benthamiana*, *Phytophthora capsici* was selected to simulate pathogen infection on *N. benthamiana*. Two days after agro‐infiltration, we inoculated *P. capsici* onto *N. benthamiana* leaves (Figures 5g and S4b). Forty‐eight hours after inoculation with PB‐GFP control and PB‐CsSP1 constructs, all the *N. benthamiana* leaves exhibited disease symptoms of *P. capsici* (Figure 5h). The control PB‐GFP plants exhibited typical and severe disease symptoms, while most lesions observed on the PB‐CsSP1 plants were small and constrained (Figures 5h and S4b). These results demonstrated that CsSp1 could trigger immunity in *N. benthamiana*. To investigate whether CsSp1 triggers plant immunity via the nucleus or cytoplasm, a nuclear localization signal (NLS) or a nuclear export signal (NES) sequence was added. At the same time, we added a mutated nuclear export signal (mNES) as a control. We constructed three protein fusions, namely CsSP1‐NLS‐GFP, CsSP1‐NES‐GFP, and CsSP1‐mNES‐GFP. As predicted, the GFP fluorescence signal was concentrated mainly in the nucleus with expression of CsSP1‐NLS‐GFP in *N. benthamiana*, while CsSP1‐NES‐GFP fluorescent signals were not concentrated in the nucleus but were localized in the cytoplasm (Figure 6a). Like CsSP1‐GFP, CsSP1‐mNES‐GFP was localized in both the nucleus and the cytoplasm (Figure 6a). All three mutant constructs were expressed normally in *N. benthamiana* (Figure 6b). We then inoculated *P. capsici* onto the leaves of *N. benthamiana* plants expressing the above three mutant constructs, and PB‐GFP was used as a control (Figure 6c). The control GFP plants exhibited typical and severe disease symptoms, while most lesions observed on CsSP1‐NLS‐GFP, CsSP1‐NES‐GFP, and CsSP1‐mNES‐GFP plants were small and constrained (Figure 6d). Taken together, these data suggest that CsSp1 triggers plant immunity in both the nucleus and the cytoplasm of *N. benthamiana* cells.

### 2.7 CsSp1 increased the expression levels of genes involved in the SA pathway

**FIGURE 7:**
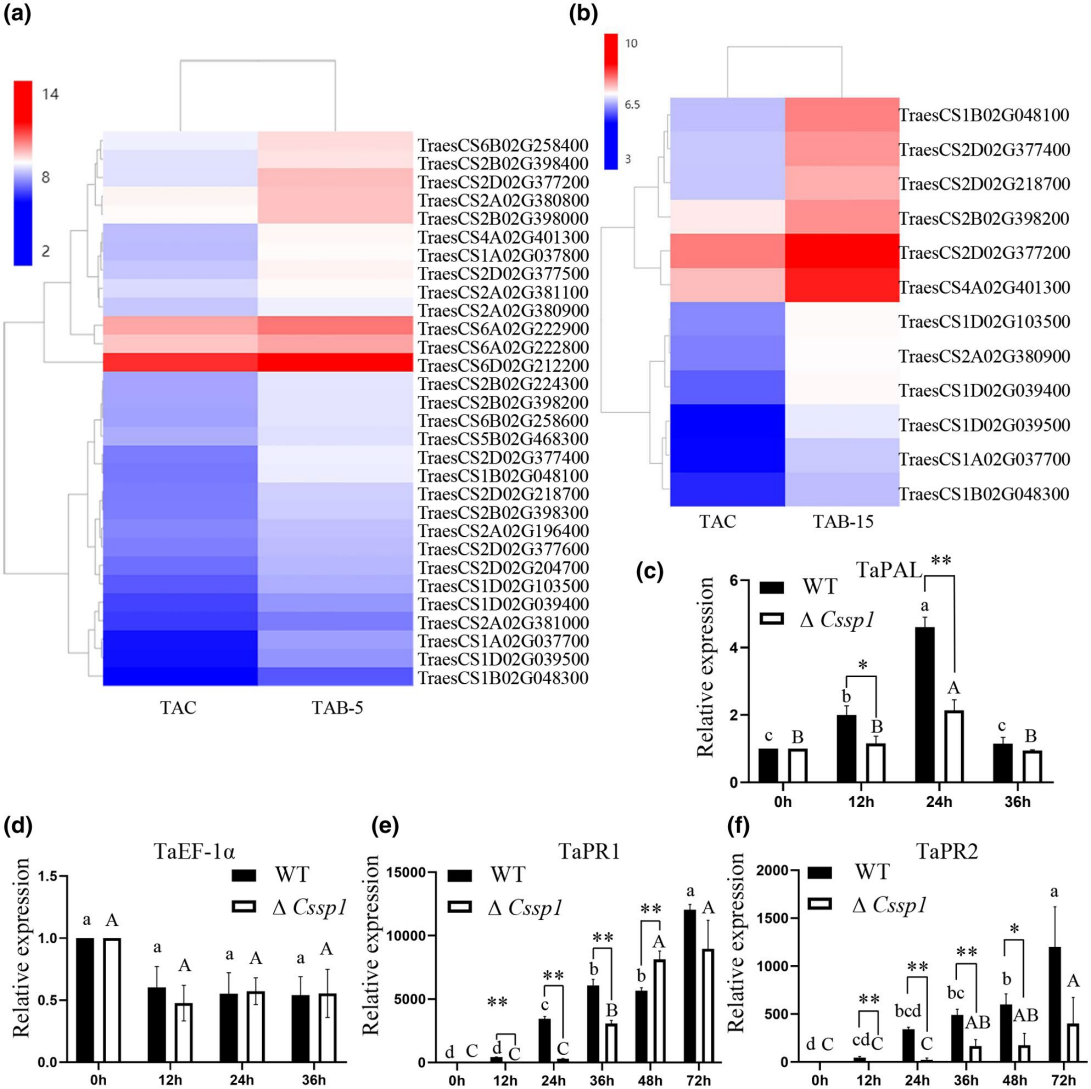
Wheat transcriptome analysis after *Bipolaris sorokiniana* infection. (a) Heatmap of differentially expressed *TaPAL*‐related genes in wheat. TAC, uninfected wheat; TAB‐5, wheat infected for 5 days. (b) Heatmap of *TaPAL* gene expression profiles. TAC, uninfected wheat; TAB‐15, wheat infected for 15 days. (c) Reverse transcription quantitative PCR (RT‐qPCR) analysis of *TaPAL* in wheat leaves infected with spores. The experiments were repeated three times. (d) Relative expression levels of constitutive *EF1α* gene during infection time course. The same letters indicate the no significant difference at *p* < 0.05 level. (e, f) RT‐qPCR for *TaPR1* and *TaPR2*, respectively, of wheat leaves infected with spores. Significant differences of samples with the same spore infection calculated by Tukey's LSD, *p* < 0.05. The lower case letters refer to a significant difference between the wild‐type (WT) samples and upper case letter refers to a significant difference between the ∆*Cssp1* samples. The significance between samples of the same infection period was tested by *t* test. **p < 0.01, *p < 0.05 (*t* test). The experiments were repeated three times. The expression levels were calculated using the 2^−∆∆^ *^C^* ^t^ method

RNA‐Seq analysis of wheat cultivar Aikang 58 infected with *B. sorokiniana* showed that the expression levels of 36 phenylalanine ammonia‐lyase (PAL) genes were significantly up‐regulated in wheat infected with *B. sorokiniana* for 5 days compared with uninfected wheat (Figure 7a). Similarly, the expression levels of 12 PAL genes were significantly up‐regulated in wheat infected with *B. sorokiniana* for 15 days compared with uninfected wheat (Figure 7b). Moreover, we verified the expression of the *TaPAL* gene, which is involved in the PAL synthesis pathway in wheat. After the wheat leaves were infected with a 10^5^/ml spore suspension, the expression level of *TaPAL* in plants inoculated with the ∆*Cssp1* strain was significantly lower than that with the WT strain (Figure 7c). To determine if the differences observed in Figure 7c are significantly different, we measured the expression of a constitutive gene. The expression level of *EF1α* in plants did not show significant differences (Figure 7d). PAL is the key enzyme in the SA synthesis pathway. After wheat leaves were infected with a 10^5^/ml spore suspension, the expression of PR genes *TaPR1* and *TaPR2* during ∆*Cssp1* infection was down‐regulated compared with that in the WT (Figure 7e,f). These findings suggest that CsSp1 interferes with the SA pathway in wheat and triggers an immune response.

## 3 DISCUSSION

Secreted effector proteins play indispensable roles in interactions between plants and phytopathogenic fungi (Giraldo & Valent, 2013). By investigating the *B. sorokiniana* transcriptome during different stages of infection (0, 5, and 15 days after inoculation) in wheat, we identified four candidates encoding putative secreted proteins. We identified one of them, CsSp1, which encodes an effector and is an essential factor for *B. sorokiniana* interactions with wheat and barley. CsSp1 predominantly targets the host nucleus and cytoplasm. Transient expression of *CsSp1* in the leaves of the model plant species *N. benthamiana* did not induce cell death but limited the development of *P. capsici*. CsSp1 was the first effector protein reported in *B. sorokiniana* to contribute an enhanced immune response and vegetative growth, sporulation, and invasive growth.

There are highly homologous *CsSp1* genes in other *Bipolaris* species, such as *Bipolaris zeicola*, *B. victoriae*, and *B. maydis*. However, the function of CsSp1 has not been studied in other *Bipolaris* fungi. Orthologous genes of *CsSp1* may play important roles in *Bipolaris* species. The pathogenicity‐related effector protein in *S. sclerotiorum*, *SsCP1*, was also expressed at the highest level at 12 hpi and remained at a high level for at least 48 hpi. *S. sclerotiorum* SsCP1 induces cell death via the host immune response, while CsSp1 does not cause cell death (Yang et al., 2018).

In *Metarhizium acridum*, deletion of *MaPMT1* does not affect appressorium formation but significantly decreases appressorium turgor pressure to weaken virulence (Wen et al., 2020). In *M. oryzae*, mutant strains lacking the spermine synthase‐encoding gene *SPS1* progress through all stages of appressorial development, including penetration peg formation, but cuticle penetration is unsuccessful due to reduced appressorial adhesion, which leads to solute leakage (Rocha et al., 2020). ∆*Cssp1* formed an abnormal appressorium, and infectious hyphal swelling indicated restricted extension in plant cells. Branching vegetative hyphae also exhibited swelling on PDA plates, and bent tip hyphae occurred on onion epidermal cells. On PDA, *B. sorokiniana* usually easily produces abundant spores (Guo, 2016). We observed a decrease in spore production by ∆*Cssp1*. Despite the decreased size of the ∆*Cssp1* spores, they could still germinate. The profile of sporulation is highly regulated, and most of the key regulators are conserved throughout filamentous fungi (Chung et al., 2011; Park & Yu, 2012; Zhao et al., 2015). We identified three key regulators, the orthologous genes *CsBrlA*, *CsMedA*, and *CsStuA*, in *B. sorokiniana*, and their expression levels significantly decreased at 24 h after cultivation. Therefore, CsSp1 is essential for vegetative development and asexual reproduction.

In hemibiotrophic fungi and oomycetes, in the early biotrophic stage, suppression of the host immune response, including the HR of programmed cell death (PCD), is suppressed. The reduced pathogenicity of ∆*Cssp1* with pleotropic defects indicates that CsSp1 is a virulence factor. In planta, the expression of CsSp1 limited the development of *P. capsici*. We concluded that this gene may enhance the immune response of plants and thus inhibit *P. capsici* infection. This elimination of plant immune responses from CsSp1 did not activate the HR of *N. benthamiana*. Wang et al. (2011) screened 169 RXLR effectors from the oomycete *P. sojae* in *N. benthamiana*. Among them, 107 effectors suppressed PCD and/or pathogen‐associated molecular pattern (PAMP)‐associated INF1 triggered by BAX. Most early effectors suppressed INF1‐triggered cell death. Similar phenomena of suppressing plant cell death by the effectors of the hemibiotrophic and necrotrophic fungal pathogens *M. oryzae* (Dong et al., 2015), *Colletotrichum orbiculare* (Yoshino et al., 2012), *Fusarium oxysporum* (Gawehns et al., 2014), and *Valsa mali* (Li et al., 2015) have also been reported. Nonetheless, the mechanism by which *B. sorokiniana* suppresses the plant immune response needs to be determined.

CsSp1 is a secreted protein with a functional signal peptide, as confirmed in a YTK12 yeast system. CsSP1‐GFP accumulated strongly in the nucleus in the presence of an additional cytoplasmic location. CsSp1 is a translocated effector that functions in the nucleus and cytoplasm of plants. There are no NLSs or other organelle localization signals in CsSp1, so the localization of CsSp1 may be influenced by the target protein. It is possible that CsSp1 target proteins are present in both the nucleus and the cytoplasm of plants. This is similar to the PSTG_23616 and UfRTP1 effector proteins and AvrM effectors of the wheat stripe rust causal agents *Uromyces fabae* and *Melampsora lini*. PSTG_23616 is distributed throughout the cell, including the nucleus, cytoplasm, and cell membrane (Kemen et al., 2005; Rafiqi et al., 2010; Song et al., 2016). Unexpectedly, there was no fluorescence in the leaves expressing CsSP1‐NS‐GFP. The presence of signal peptides may protect the proteins from being easily degraded. We used PSORT (https://www.genscript.com/psort.html) to predict that CsSp1 has a cleavable signal peptide (1–15 amino acids). The results were not the same as the predicted ones shown in Figure 5a. We used YinOYang 1.2 Server (http://www.cbs.dtu.dk/services/yinoyang/) to predict O‐glycosylation sites, six of which were identified in CsSp1. N16 was predicted to be a glycosylation site by YinOYang 1.2 Server (Table 1). In our study, N16 was deleted as part of the signal peptide. Removing two additional amino acid residues (in which one is a potential glycosylation site) might cause the mature protein to be unstable. N‐glycosylation shields the *P. sojae* apoplastic effector PsXEG1, which is sensitive to a specific host aspartic protease (Xia et al., 2020), so we hypothesize that glycosylation of CsSp1 may also affect protein degradation by some proteases. This did not affect the localization of CsSP1‐NS‐GFP in the inner epidermis of onions, and the stability of protein expression might be different in different plant species. Further research on the subcellular localization of CsSp1 and signal peptide function is needed. Through *Agrobacterium*‐mediated transient expression analysis, CsSp1 was shown to stimulate an immunoreaction in *N. benthamiana* to suppress *P. capsici* colonization. In contrast, the expression of the *U. virens* effector UVSix1‐1 in *N. benthamiana* could inhibit cell death and promote the colonization of *P. capsici* (Li et al., 2019). Many effectors that function in the nucleus to regulate plant immunity have been reported. The *Verticillium*‐specific protein VdSCP7 localizes to the nucleus of plant cells and modulates immunity to fungal infection (Zhang et al., 2017).

Plant hormones, including the classic ones SA, JA, and ethylene (ET), regulate the defence response to various pathogens (Kazan & Lyons, 2014). SA is essential for modulating the regulatory response to biotrophic and hemibiotrophic pathogens (Han & Kahmann, 2019). It is widely known that *B. sorokiniana* mainly triggers the SA signalling pathway, but the underlying mechanism remains unknown (Aldaoude, 2019). In our study, compared with uninoculated leaves, *B. sorokiniana*‐inoculated leaves (at the early stage) presented a substantial amount of PAL activity (Mali et al., 2017; Singh et al., 2019). This is consistent with our findings that the expression of *TaPAL* was up‐regulated in the infection stage and that the expression of *TaPAL* in ∆*Cssp1* cells was lower than that in WT cells. In *S. sclerotiorum*, the virulence factor SsCP1 indeed interacts directly with the PR protein PR1 in planta (Yang et al., 2018). Moreover, SA‐dependent genes including PR1, PR2, and PR5 were found to be up‐regulated in 35S:Sscp1 transgenic *A. thaliana* lines (Yang et al., 2018). In addition, RNA interference assays showed that infection with the *BxSapB1‐*silenced nematode *Bursaphelenchus xylophilus* resulted in significantly decreased expression of the PR genes *PtPR‐1b*, *PtPR‐3*, and *PtPR‐5*, and delayed the onset of symptoms in pine seedlings (Hu et al., 2019). These findings were verified by our results in which CsSp1 was shown to trigger the SA pathway of *B. sorokiniana–*wheat interactions. Triggering of the SA pathway by CsSp1 without inducing host cell death improves our understanding of wheat challenged with *B. sorokiniana* and/or *Bipolaris* spp.

Collectively, this study provides information on some functional features of CsSp1, paving the way for an improved understanding of the molecular mechanism through which pathogen effectors manipulate the plant immune system.

## 4 EXPERIMENTAL PROCEDURES

### 4.1 Plant materials and fungal strains

The susceptible wheat variety Aikang 58 was used for bioassays. *B. sorokiniana* Lankao 9‐3 and pYIP‐102‐3FLAG and pKOV21 vectors were maintained in our laboratory. *N. benthamiana* and the PB‐HA and PVX‐3HA vectors were kindly provided by Dr Yuanchao Wang from Nanjing Agricultural University. The yeast strain YTK12 and the pSUC‐Avr1b and pSUC vectors were generously provided by Dr Xiaojie Chen from Northwest Agriculture & Forestry University.

### 4.2 Gene knockout

The split‐marker gene knockout strategy was used in this study according to the description of Wang et al. (2017), with slight modifications. Briefly, for homologous recombination fragments, upstream and downstream fragments of the target gene (approximately 1 kb) were amplified from the genomic DNA of *B*. *sorokiniana* Lankao 9‐3 via primer pairs CsSP1‐F1/CsSP1‐R1 and CsSP1‐F2/CsSP1‐R2. Split hygromycin B resistance gene fragments were generated from pKOV21 plasmid DNA. The three fragments were fused together via PCR homologous recombination. Next, using the fusion fragment as the template, the upstream region of the target gene and the front two‐thirds of the hygromycin resistance gene sequence were amplified, after which the back two‐thirds of the hygromycin resistance gene sequence and the downstream region of the target gene were amplified. Two fragments were prepared for protoplast transformation. The amplified fragments were purified using B518141‐0100 (SanPrep Column PCR Product Purification Kit) from Sangon Biotech.

The protoplast preparation methods were based on those for *Fusarium graminearum* (Wang et al., 2012).

### 4.3 Fungal growth and sporulation

The spores of the original WT strain Lankao 9‐3 in glycerol were kept in a freezer at −80°C. Cultures for fungal growth, sporulation, and pathogenicity tests started from this stock. A 5‐mm fungal block was shredded with a blender and then placed on 15 ml of PDA (200 g peeled potato, 20 g dextrose, 20 g agar, 1 L water), which was placed in an incubator at 25°C under darkness for 7 days. The diameter of colony growth was measured after 7 days. The hyphae were then removed and cultured under light for 5 days, and the spores were washed with 5 ml of double deionized water and counted. The experiment involved three replicates. Before the spores were inoculated onto barley and wheat, we first cultured them in darkness for 24 h and then in light for 2–3 days. The mycelial preparation was performed in YEPD (1% yeast extract, 2% Bacto peptone, 2% dextrose) broth.

For solophenyl flavine 7GFE fluorescent dye staining, barley seedlings of the Kenpimai 7 cultivar were prepared as described by Zhang, Wang, et al. (2020), with slight modifications. Briefly, a 5‐mm diameter fungal culture plug from a PDA plate (cultured for 3–4 days) was placed on the barley leaves. The tray was sealed with plastic wrap and kept in a moist box at 25°C for 24 h in the dark, after which time it was moved to a greenhouse at 25°C (47% humidity) with a 16 h light/8 h dark photoperiod. After 3 days, the barley leaves were collected, placed in 95% ethanol for 48 h and then immersed in boiling water for 15 min for discolouration. Before being cleared in 50 mM NaOH twice for 15 min each, the leaves were rinsed with 50% ethanol for 15 min. After washing with water three times for 10 min each, the samples were transferred to 0.1 M Tris‐HCl (pH 8.5) for 30 min, after which point the samples were ready for staining or kept in the solution. A stock solution of solophenyl flavine 7GFE (0.1% wt/vol) was diluted 50 times with Tris‐HCl buffer (pH 8.5) to prepare a working solution. The cleared barley leaves were stained for 5 min in the working solution and washed twice with double deionized water. The stained leaf tissues were subsequently cut into small pieces and placed on glass microslides with coverslips for microscopy.

For infection of onion inner epidermis, the third to fifth layers of the onion inner epidermis were removed with a sharp blade, and the concave surface of the onion inner epidermis was spread upside down in the orifice of a 24‐well plate containing sterile water. A spore solution (20 μl, 5 × 10^4^/ml) was dropped onto the onion epidermis. The cells were placed in a 25°C illuminated humid chamber, and the structure of the appressoria was observed via microscopy after inoculation.

For data analysis, the standard deviation was used for all errors throughout the experiments. Significant differences were calculated by Tukey's LSD, *p* < 0.05 and *t* test.

### 4.4 RNA‐Seq and data analysis

The details of Aikang 58 wheat seedlings infected with or without *B. sorokiniana* WT strain Lankao 9‐3 in pots were described by Kang et al. (2020). Seedling samples were carefully collected at 5 and 15 days after inoculation and rinsed with tap water to assess disease development. The same method was used to harvest samples from the field. The collected clean stem bases and roots were subsequently frozen in liquid nitrogen and kept at −80°C until use. We harvested spores growing on PDA plates for 7 days, filtered them through a layer of sterile Miracloth (Millipore Corp.), collected them via centrifugation at 1000 × *g* for 5 min and then washed them twice with sterile distilled water. Afterwards, 250‐ml flasks were inoculated with solutions (10^6^ spores/ml) containing 100 ml YEPD broth. After 3 days of shaking at 150 rpm at 25°C, mycelia were collected through sterile Miracloth filters into a funnel and washed twice with sterile distilled water. Extra water on the absorbent paper was removed, after which the mycelia were frozen in liquid nitrogen and stored at −80°C. The wheat and mycelial samples (two replicates each) were subsequently sent to Novogene (Tianjin Novogene Bioinformatic Technology Co., Ltd). RNA and library qualities were assessed via a Bioanalyzer 2100 system (Agilent Technologies). Principal component analysis was used to analyse the similarity of RNA‐Seq samples (Figure S2a,b). The library was sequenced on an Illumina NovaSeq platform (HiSeq 2500), and 150 bp paired‐end reads were generated. The clean paired‐end reads were then aligned to the *B. sorokiniana* genome (https://mycocosm.jgi.doe.gov) using HISAT v. 2.0.5. Differential expression analysis with an adjusted *p* value of <0.05 between treatments was performed using the DESeq2 R package v. 1.20.0. The fragments per kilobase of transcript per million mapped reads (FPKM) of each gene was calculated based on the length of the gene and reads count mapped to that gene. The cluster Profiler R package was used for Gene Ontology (GO) and Kyoto Encyclopedia of Genes and Genomes (KEGG) enrichment analysis of differentially expressed genes (DEGs).

### 4.5 Construction of expression vectors

For complementation of the knockout mutant, the target gene *CsSP1* ORF with a 1.8 kb promoter region and without the stop codon was amplified from *B. sorokiniana* strain Lankao 9‐3 genomic DNA. The primer pair pYIP‐CsSP1‐F/pYIP‐CsSP1‐R was designed to introduce *Pst*Ⅰ sites at the 5ʹ and 3ʹ ends. The fragment amplicons were subsequently cloned and ligated into a pYIP‐102 expression vector at the *Pst*Ⅰ cleavage site. The positive clone confirmed by sequencing was then transformed into protoplasts of the ∆*Cssp1* deletion mutant.

For expression in *Agrobacterium tumefaciens*, the target gene *CsSP1* ORF without a stop codon was amplified from *B. sorokiniana* strain Lankao 9‐3 complementary DNA. The primer pair PB‐CsSP1‐F/PB‐CsSP1‐R was designed to introduce a *Bam*HI site and a *Kpn*I site at the 5ʹ and 3ʹ ends, respectively. The synthesized foreign gene expression cassette was inserted into a PB‐HA *Agrobacterium* expression vector and then cloned into *Escherichia coli* Top10 cells by the heat shock method. First, the fragment and the linear vector were fused with a one‐step cloning kit (Vazyme Biotech). Briefly, the product was added to 50 µl of *E. coli* cells. PVX and pSUC2 vectors were constructed in a similar way.

For GFP fusion, the target gene *CsSP1* ORF without a stop codon was amplified from *B. sorokiniana* strain Lankao 9‐3 complementary DNA. Primer pairs were designed to introduce homologous segments of GFP, and then the GFP fragment was fused by homologous recombination. For construction without a signal peptide, primer pairs were designed for amplification without the signal peptide.

### 4.6 Polyacrylamide gel electrophoresis and western blotting

For protein extraction, mycelial or plant samples were ground to a fine powder in liquid nitrogen. The fine powder was then treated with protein extraction buffer (20 mM Tris‐HCl pH 7.5, 150 mM NaCl, 1 mM EDTA, 20% glycerol, 1 mM phenylmethylsulfonyl fluoride [PMSF], and a suitable concentration of protease inhibitor). After swirling and mixing, the proteins were released after 10 min in an ice bath. After centrifugation at 13,000 × *g* for 15 min, the supernatant, which contained the desired protein, was transferred to a new centrifuge tube. The mixture was brought to 5 ml with double deionized water and mixed well, with β‐mercaptoethanol added just before use. After immersion in boiling water for 10 min, the sample was used for protein extraction.

For western blotting, the methods were performed according to those of Li et al. (2018), and anti‐HA antibodies (M20003) were used (http://www.ab‐mart.com/).

### 4.7 Agro‐infiltration assays

The PB‐based (or PVX‐based) *CsSP1* gene constructs were transformed into *A. tumefaciens* GV3101 through heat shock transformation. The *A. tumefaciens* clones containing *CsSP1* constructs were cultured in liquid LB medium (10 g tryptone, 10 g NaCl, 5 g yeast extract, and 950 ml double deionized water) supplemented with rifampicin (50 μg/ml) and kanamycin (50 μg/ml). The *A. tumefaciens* cultures were collected by centrifugation, washed with MMA buffer (10 mM 2‐[*N*‐morpholino] ethanesulfonic acid ([MES; pH 5.7], 10 mM MgCl_2_, 150 μM acetosyringone), and then resuspended in agro‐infiltration MMA buffer at an OD_600_ of 0.5 at room temperature for 2 h. Agro‐infiltration experiments were carried out on the leaves of 4‐week‐old *N. benthamiana* plants using needleless syringes. The inner epidermis of the third to fifth layers of onion was treated with the same concentration of *Agrobacterium* for 20 min and then placed on solid MA medium (10 mM MgCl_2_, 150 μM acetosyringone) at 25°C under 16 h of light and 8 h of darkness. Fluorescence was observed after treatment for 24 h. The inoculation method for the inner epidermis of onions was the same as that reported by Liu et al. (2009), and the inoculation method for *P. capsici* was the same as that of Li et al. (2019).

### 4.8 Yeast secretion assays

A pSUC2 vector containing a tryptophan synthesis‐related gene but lacking a signal peptide, as well as the invertase gene (*SUC2*) without the start codon (ATG), was used. The product of the invertase gene (*SUC2*) converts polysaccharides to monosaccharides. Therefore, only when the secreted gene is inserted can the missing *SUC2* gene be activated and secreted into the culture medium for the conversion of sucrose to glucose needed for yeast growth. The methods used refer mainly to the protocols described previously (Gu et al., 2011; Li et al., 2016, 2019). We integrated these approaches and made several modifications. First, 10% dimethyl sulphoxide (DMSO) was added to the yeast competent cells. In addition, sucrose selective medium (SD−Trp−sucrose medium, 0.8 g yeast synthetic drop‐out medium without Trp, 2% sucrose, 2% agar) was used for the final screening (Guo, Zhong, et al., 2019).

### 4.9 Gene expression analysis

Total RNA for measuring *CsSP1* expression was extracted from *B. sorokiniana* mycelia grown for 2 days in YEPD medium and wheat leaves inoculated with 10^5^ spores/ml. To measure gene expression during sporulation, total RNA was extracted from *B. sorokiniana* mycelia in PDB medium, and to measure the expression of *PAL*, *PR1*, and *PR2*, total RNA was extracted from wheat leaf samples with or without inoculation of 10^5^ spores/ml.

An RN38‐EASY Spin Plus Plant RNA Kit (Aidlab Biotechnologies Co., Ltd) was used to extract total RNA from *B. sorokiniana* mycelia or wheat leaves. One microgram of total RNA was used for reverse transcription using a PrimeScript RT Reagent Kit with gDNA Eraser (Perfect Real Time). The transcript levels of *CsSP1* were measured via RT‐qPCR under standard conditions with the gene‐specific primers CsSP1‐QRT2‐F/CsSP1‐QRT2‐R. The expression level of *β‐actin*, as an internal reference gene, in *B. sorokiniana* in conjunction with the primer pair RT‐actin‐F/RT‐actin‐R was measured (Wang et al., 2015). The expression of candidate genes *CsBrlA*, *CsMedA*, and *CsStuA* of *Aspergillus nidulans*, which are *BrlA*, *MedA*, and *StuA* orthologs in *B. sorokiniana*, in conjunction with the primer pairs CsBrlA‐F/CsBrlA‐R, CsMedA‐F/CsMedA‐R, and CsStuA‐F/CsStuA‐R, respectively, was measured (Wang et al., 2015).

All the internal reference primers used to express the wheat genes were Actin‐PR‐F/Actin‐PR‐R (Naz et al., 2018). The primers used to detect *TaPAL* were TaTAL‐F/TaPAL‐R, while those used to detect *TaPR1* and *TaPR2* were TaPR1‐F/TaPR1‐R and TaPR2‐F/TaPR2‐R (Niu et al., 2007). All the primers used are listed in Table 2. The relative transcript levels of test genes were determined according to the function ∆*C*_t_ = *C*_t_ (test gene) − *C*_t_ (reference gene). Briefly, the threshold cycles (*C*_t_) of the PCR results for each gene were first obtained and then averaged for use of quantification of the transcripts in the next step. The ∆*C*_t_ value was determined by subtracting the average *C*_t_ value of the endogenous reference genes, *Actin* or *EF1α* in this study, from the average *C*_t_ value of the candidate gene. The ∆∆*C*_t_ value was calculated by subtracting the ∆*C*_t_ value of the *B. sorokiniana* mycelia control from the ∆*C*_t_ of the inoculated sample. The 2^−∆∆^ *^C^* ^t^ value was used to evaluate the fold change of gene expression. The relative expression under different conditions was calculated according to the 2^−ΔΔ^ *^C^* ^t^ method (Livak & Schmittgen, 2001). Each experiment had three replicates to give the main value and the standard deviations were generated.

**TABLE 2:** The primers used in the experiment

Primer	Sequence 5′–3′	Purpose
CsSP1‐F1	GAGGTCGTGGGACTATCTTATAGCCAG	Amplification of upstream fragment of *CsSP1*
CsSP1‐R1	TTGACCTCCACTAGCTCCAGCCAAGCCTTGAAGTCCGGTACGCTGTGAATGAA	
CsSP1‐F2	ATAGAGTAGATGCCGACCGCGGGTTCCTAGCAATACCAGATGTAAC	Amplification of downstream fragments of *CsSP1*
CsSP1‐R2	GATCCCATGGCCTGCTCTAAGA	
CsSP1‐NF	GTGGCTTCCGCACCTGCTACA	Amplification of internal fragment of *CsSP1*
CsSP1‐NR	CTTCTCCGACCCTGGATATTG	
CsSP1‐PF	TTAGCCGCAGATGACCTTG	Generation of knockout mutant
CsSP1‐PR	CCGCAAGTGCATGGGTGTAAG	
HYG‐F	GGCTTGGCTGGAGCTAGTGGAGGTCAA	Amplification of *hygromycin* *resistance* gene
HY‐R	GTATTGACCGATTCCTTGCGGTCCGAA	
YG‐F	GATGTAGGAGGGCGTGGATATGTCCT	
HYG‐R	GAACCCGCGGTCGGCATCTACTCTAT	
H855R	GCTGATCTGACCAGTTGC	
H856F	GTCGATGCGACGCAATCGT	
PVX‐CsSP1‐F	CACCAGCTAGCATCGATTCCCGGG ATGCGTTTCATCATCTCC	Construction of PVX carrier
PVX‐CsSP1‐NS‐F	CACCAGCTAGCATCGATTCCCGGGATGGAGGTGGCTTCCGCA	
CsSP1‐GFP‐R	CTCGCCCTTGCTCACCATGGTCCCGCGGTTTGGC	
GFP‐CsSP1‐F	GCCAAACCGCGGGACCATGGTGAGCAAGGGCGAG	
PVX‐GFP‐F	CACCAGCTAGCATCGATTCCCGGGATGGTGAGCAAGGGCGAG	
PVX‐GFP‐R	CGCAATCTCTAGAGGATCCTTGTACAGCTCGTCCAT	
CsSP1‐PYIP‐F	GCTTGATATCGAATTCCTGCAGGATCATTTCCATCATCGCGCT	Construction of PYIP carrier
CsSP1‐PYIP‐R	GAAAATAAAGATTCTCGGTCCCGCGGTTTGGCTGTTC	
PB‐CsSP1‐F	CATTTACGAACGATAGGGTACCATGCGTTTCATCATCTCCATATTC	Construction of PB carrier
PB‐CsSP1‐R	GGTAAGGCCTACTAGTGGATCCGGTCCCGCGGTTTGGCTGTTC	
pSUC‐CsSP1‐F	AAGCTCGGAATTTTAATTAAATGCGTTTCATCATCTCCATA	Construction of pSUC carrier
pSUC‐CsSP1‐R	CGACTCACTATAGGGAGAACGGTCCCGCGGTTTGGCTGTTC	
CsSP1‐QRT2‐F	CAGCTCTTGCATCTGCTA	Expression of CsSP1
CsSP1‐QRT2‐R	TCTTCTCCGACCTTGAG	
RT‐Actin‐F	GTATGGGCCAAAAGGACTCA	Internal reference primers of *B. sorokiniana*
RT‐Actin‐R	CACGCAGCTCGTTGTAGAAG	
TaPR1‐F	CTGGAGCACGAAGCTGCAG	Expression of *PR* gene in wheat
TaPR1‐R	CGAGTGCTGGAGCTTGCAGT	
TaPR2‐F	CTCGACATCGGTAACGACCAG	
TaPR2‐R	GCGGCGATGTACTTGATGTTC	
Actin‐PR‐F	GGAAAAGTGCAGAGAGACACG	Internal reference primers from TaBs109G1 clone of wheat
Actin‐PR‐R	TACAGTGTCTGGATCGGTGGT	
TaPAL‐F	TTCGATTTGCCACCAAGTC	Expression of *PAL* gene in wheat
TaPAL‐R	GTGCCTTGGAAGTTGCCAC	
CsBrlA‐F	TCCCGAAGCTATGGTGGATC	Expression of conidiation‐related genes
CsBrlA‐R	CTGGCTGGAGGTTGATGGTA	
CsMedA‐F	TGGTGGGAAGAAAAGGACGA	
CsMedA‐R	TCGCTGTCTTGCTTTGCTTT	
CsStuA‐F	ACCCAAAGACAGAGATGGCA	
CsStuA‐R	ACTCATTGCCGTGATCATGC	
CsSP1‐NLS‐F	GCCAAACCGCGGGACCCAGCCTAAGAAGAAGAGAAAGGTTGGAGGAATGGTGAGCAAGGGCG	Construction of CsSP1 location‐based signal mutants
CsSP1‐NES‐F	GCCAAACCGCGGGACCAACGAGCTTGCTCTTAAGTTGGCTGGACTTGATATTAACAAGATGGTGAGCAAGGGCG	
CsSP1‐mNES‐F	GCCAAACCGCGGGACCATGCTTCAAGCTCCTCCTGCTGAAAGAGCTACTCTTATGGTGAGCAAGGGCG	
PB‐GFP‐R	GGTAAGGCCTACTAGTGGATCCCTTGTACAGC TCGTCCATGC	
TaEF‐1α‐F	CAAGGGTGTGGAGAAGAAGG	Expression of a constitutive gene of wheat
TaEF‐1α‐R	AGCAGACATAGATGGATTCAGG	

## Supporting information


**FIGURE S1** Root rot of wheat caused by *Bipolaris sorokiniana* (the samples of which were used for transcriptome sequencing). (a, b) Seedlings and roots at 5 days and (c, d) 15 days after inoculation. Mock, wheat without inoculation; B.s, inoculation with *B. sorokiniana*

**FIGURE S2** (a) Principal component analysis (PCA) of RNA‐Seq data of *Bipolaris sorokiniana*. (b) Principal component analysis (PCA) of RNA‐Seq data of *Triticum aestivum*. (c) Phylogenetic tree of orthologues of CsSp1 generated through MEGA 7. Bar, 0.1

**FIGURE S3** (a) Pathogenicity test with millet inoculum of *∆Cssp1* and the wild‐type (WT) strain applied to the soil. (b) Three days after inoculation of wheat coleoptiles with agar blocks of fungal cultures. (c) Spore germination and appressorium formation of *∆Cssp1* on onion epidermal cells compared with WT cells. (d) The rate of normal appressorium production

**FIGURE S4** (a) Subcellular localization of CsSp1‐GFP in H2B‐red transgenic *Nicotiana benthamiana* leaves. Bar, 20 μm. ① The signal peaks of CsSP1 green and H2B red obviously merged together. The white arrow shows the obvious co‐location site. (b) Independent replicates of those shown in Figure 5g

**FIGURE S5** (a) Prediction of CsSp1 O‐glycosylation sites via YinOYang 1.2. (b) Prediction of CsSp1 phosphorylation sites via NetPhos 3.1a. The threshold was 0.6

## Data Availability

The RNA‐Seq data are available from GenBank at https://www.ncbi.nlm.nih.gov/genbank/ with accession BioProject PRJNA743515.

## References

[mpp13155-bib-0001] Acharya, K. , Dutta, A.K. & Pradhan, P. (2011) *Bipolaris sorokiniana* (Sacc.) Shoem. The most destructive wheat fungal pathogen in the warmer areas. Australian Journal of Crop Science, 5, 1064–1071.

[mpp13155-bib-0002] Aldaoude, A. (2019) Transcriptional changes of salicylic acid dependent signaling pathways in barley–*Cochliobolus sativus* interaction. Journal of Plant Biochemistry & Physiology, 7, 228.

[mpp13155-bib-0003] Al‐Daoude, A. , Al‐Shehadah, E. , Shoaib, A. , Jawhar, M. & Arabi, M.I.E. (2018) Salicylic acid and hydrogen peroxide accumulation in relation to hydrolyte leakage in barley plants challenged with *Cochliobolus sativus* . Cereal Research Communications, 46, 650–657.

[mpp13155-bib-0004] Alvarez, M.E. (2000) Salicylic acid in the machinery of hypersensitive cell death and disease resistance. Plant Molecular Biology, 44, 429–442. 11199399 10.1023/a:1026561029533

[mpp13155-bib-0005] Anand, A. , Schmelz, E.A. & Muthukrishnan, S. (2003) Development of a lesion‐mimic phenotype in a transgenic wheat line overexpressing genes for pathogenesis‐related (PR) proteins is dependent on salicylic acid concentration. Molecular Plant‐Microbe Interactions, 16, 916–925. 14558693 10.1094/MPMI.2003.16.10.916

[mpp13155-bib-0006] Anderson, R.G. , Casady, M.S. , Fee, R.A. , Vaughan, M.M. , Deb, D. , Fedkenheuer, K. et al. (2012) Homologous RXLR effectors from *Hyaloperonospora arabidopsidis* and *Phytophthora sojae* suppress immunity in distantly related plants. The Plant Journal, 72, 882–893. 22709376 10.1111/j.1365-313X.2012.05079.x

[mpp13155-bib-0007] Boller, T. & Felix, G. (2009) A renaissance of elicitors: perception of microbe‐associated molecular patterns and danger signals by pattern‐recognition receptors. Annual Review of Plant Biology, 60, 379–406. 10.1146/annurev.arplant.57.032905.105346 19400727

[mpp13155-bib-0008] Bourras, S. , Mcnally, K.E. , Ben‐David, R. , Parlange, F. & Keller, B. (2015) Multiple avirulence loci and allele‐specific effector recognition control the *Pm3* race‐specific resistance of wheat to powdery mildew. The Plant Cell, 27, 2991–3012. 26452600 10.1105/tpc.15.00171 PMC4682313

[mpp13155-bib-0009] Bozkurt, T.O. & Kamoun, S. (2020) The plant–pathogen haustorial interface at a glance. Journal of Cell Science, 133, jcs237958. 10.1242/jcs.237958 PMC7075074 32132107

[mpp13155-bib-0011] Caillaud, M.‐C. , Piquerez, S.J.M. , Fabro, G. , Steinbrenner, J. & Jones, J.D.G. (2012) Subcellular localization of the Hpa RxLR effector repertoire identifies a tonoplast‐associated protein HaRxL17 that confers enhanced plant susceptibility. The Plant Journal, 69, 252–265. 21914011 10.1111/j.1365-313X.2011.04787.x

[mpp13155-bib-0012] Chung, D.‐W. , Greenwald, C. , Upadhyay, S. , Ding, S. , Wilkinson, H.H. , Ebbole, D.J. et al. (2011) acon‐3, the *Neurospora crassa* ortholog of the developmental modifier, medA, complements the conidiation defect of the *Aspergillus nidulans* mutant. Fungal Genetics and Biology, 48, 370–376. 21220038 10.1016/j.fgb.2010.12.008

[mpp13155-bib-0013] Condon, B.J. , Leng, Y. , Wu, D. , Bushley, K.E. , Ohm, R.A. , Otillar, R. et al. (2013) Comparative genome structure, secondary metabolite, and effector coding capacity across *Cochliobolus* pathogens. PLoS Genetics, 9, e1003233. 23357949 10.1371/journal.pgen.1003233 PMC3554632

[mpp13155-bib-0014] Dai, J. , Yu, Q. , Hong‐Xia, Y.H. , Xing, X. , Zhang, M. , Sun, B. et al. (2011) Isolation, identification and pathogenicity of the pathogens of wheat black point in Henan Province. Acta Phytopathologica Sinica, 41, 225–231.

[mpp13155-bib-0015] Diaz‐Granados, A. , Sterken, M.G. , Overmars, H. , Ariaans, R. , Holterman, M. , Pokhare, S.S. et al. (2020) The effector GpRbp‐1 of *Globodera pallida* targets a nuclear HECT E3 ubiquitin ligase to modulate gene expression in the host. Molecular Plant Pathology, 21, 66–82. 31756029 10.1111/mpp.12880 PMC6913204

[mpp13155-bib-0016] Djamei, A. , Schipper, K. , Rabe, F. , Ghosh, A. , Vincon, V. , Kahnt, J. et al. (2011) Metabolic priming by a secreted fungal effector. Nature, 478, 395–398. 21976020 10.1038/nature10454

[mpp13155-bib-0017] Dong, Y. , Li, Y. , Zhao, M. , Jing, M. , Liu, X. , Liu, M. et al. (2015) Global genome and transcriptome analyses of *Magnaporthe oryzae* epidemic isolate 98–06 uncover novel effectors and pathogenicity‐related genes, revealing gene gain and lose dynamics in genome evolution. PLoS Pathogens, 11, e1004801. 25837042 10.1371/journal.ppat.1004801 PMC4383609

[mpp13155-bib-0018] Gawehns, F. , Houterman, P.M. , Ichou, F.A. , Michielse, C.B. & Takken, F.L.W. (2014) The *Fusarium oxysporum* effector Six6 contributes to virulence and suppresses I‐2‐mediated cell death. Molecular Plant‐Microbe Interactions, 27, 336–348. 24313955 10.1094/MPMI-11-13-0330-R

[mpp13155-bib-0019] Ghazvini, H. & Tekauz, A. (2012) Molecular diversity in the barley pathogen *Bipolaris sorokiniana* (*Cochliobolus sativus*). Australasian Plant Pathology, 41, 283–293.

[mpp13155-bib-0020] Giraldo, M.C. , Dagdas, Y.F. , Gupta, Y.K. , Mentlak, T.A. , Yi, M. , Martinez‐Rocha, A.L. et al. (2013) Two distinct secretion systems facilitate tissue invasion by the rice blast fungus *Magnaporthe oryzae* . Nature Communications, 4, 1996. 10.1038/ncomms2996 PMC3709508 23774898

[mpp13155-bib-0021] Giraldo, M.C. & Valent, B. (2013) Filamentous plant pathogen effectors in action. Nature Reviews Microbiology, 11, 800–814. 24129511 10.1038/nrmicro3119

[mpp13155-bib-0022] Godfrey, D. , Böhlenius, H. , Pedersen, C. , Zhang, Z. , Emmersen, J. & Thordal‐Christensen, H. (2010) Powdery mildew fungal effector candidates share N‐terminal Y/F/WxC‐motif. BMC Genomics, 11, 317. 20487537 10.1186/1471-2164-11-317 PMC2886064

[mpp13155-bib-0023] Gu, B. , Kale, S.D. , Wang, Q. , Wang, D. , Pan, Q. , Cao, H. et al. (2011) Rust secreted protein Ps87 is conserved in diverse fungal pathogens and contains a RXLR‐like motif sufficient for translocation into plant cells. PLoS One, 6, e2721. 10.1371/journal.pone.0027217 PMC3208592 22076138

[mpp13155-bib-0024] Guo, H. , Yao, Q. , Chen, L. , Wang, F. & Xu, S. (2019) Virulence and molecular diversity in the *Cochliobolus sativus* population causing barley spot blotch in China. Plant Disease, 103, 2252–2262. 31298990 10.1094/PDIS-11-18-2103-RE

[mpp13155-bib-0025] Guo, X. , Zhong, D. , Xie, W. , He, Y. , Zheng, Y. , Lin, Y. et al. (2019) Functional identification of novel cell death‐inducing effector proteins from *Magnaporthe oryzae* . Rice, 12, 59. 31388773 10.1186/s12284-019-0312-z PMC6684714

[mpp13155-bib-0026] Guo, Y. (2016) Phylogenetic analysis of the genera *Bipolaris* and *Curvularia* in China. Beijing: Chinese Academy of Agricultural Sciences. (Ph.D. thesis). DOI: CNKI:CDMD:2.1016.174505.

[mpp13155-bib-0027] Gupta, P.K. , Chand, R. , Vasistha, N.K. , Pandey, S.P. , Kumar, U. , Mishra, V.K. et al. (2018) Spot blotch disease of wheat: the current status of research on genetics and breeding. Plant Pathology, 67, 508–531.

[mpp13155-bib-0028] Han, Q. , Huang, L. , Buchenauer, H. , Wang, C. & Kang, Z. (2010) Cytological study of wheat spike infection by *Bipolaris sorokiniana* . Journal of Phytopathology, 158, 22–29.

[mpp13155-bib-0029] Han, X. & Kahmann, R. (2019) Manipulation of phytohormone pathways by effectors of filamentous plant pathogens. Frontiers in Plant Science, 10, e822. 10.3389/fpls.2019.00822 PMC6606975 31297126

[mpp13155-bib-0030] Houterman, P.M. , Cornelissen, B. & Rep, M. (2008) Suppression of plant resistance gene‐based immunity by a fungal effector. PLoS Pathogens, 4, e1000061. 18464895 10.1371/journal.ppat.1000061 PMC2330162

[mpp13155-bib-0031] Houterman, P.M. , Ma, L. , Ooijen, G.V. , Vroomen, M. & Rep, M. (2010) The effector protein Avr2 of the xylem‐colonizing fungus *Fusarium oxysporum* activates the tomato resistance protein I‐2 intracellularly. Plant Journal, 58, 970–978. 10.1111/j.1365-313X.2009.03838.x 19228334

[mpp13155-bib-0032] Hu, L.J. , Wu, X.Q. , Li, H.Y. , Zhao, Q. , Wang, Y.C. & Ye, J.R. (2019) An effector, BxSapB1, induces cell death and contributes to virulence in the pine wood nematode *Bursaphelenchus xylophilus* . Molecular Plant‐Microbe Interactions, 32, 452–463. 30351223 10.1094/MPMI-10-18-0275-R

[mpp13155-bib-0034] Jiang, R.H.Y. , Tripathy, S. , Govers, F. & Tyler, B.M. (2008) RXLR effector reservoir in two *Phytophthora* species is dominated by a single rapidly evolving superfamily with more than 700 members. Proceedings of the National Academy of Sciences of the United States of America, 105, 4874–4879. 18344324 10.1073/pnas.0709303105 PMC2290801

[mpp13155-bib-0035] Kang, R. , Hu, Y. , Wang, L. , Xie, S. & Li, H. (2020) Pathogenicity variation and DNA polymorphism of *Bipolaris sorokiniana* infecting winter wheat in the Huanghuai floodplain of China. Plant Pathology, 70, 87–99.

[mpp13155-bib-0036] Karov, I. , Mitrev, S. & Kostadinovska, E. (2009) *Bipolaris sorokiniana* (teleomorph *Cochliobolus Sativus*): causer of barley leaf lesions and root rot in *Macedonia* . Zbornik Matice Srpske Za Prirodne Nauke, 116, 167–174.

[mpp13155-bib-0037] Kazan, K. & Lyons, R. (2014) Intervention of phytohormone pathways by pathogen effectors. The Plant Cell, 26, 2285–2309. 24920334 10.1105/tpc.114.125419 PMC4114936

[mpp13155-bib-0039] Kemen, E. , Kemen, A.C. , Rafiqi, M. , Hempel, U. , Mendgen, K. , Hahn, M. et al. (2005) Identification of a protein from rust fungi transferred from haustoria into infected plant cells. Molecular Plant‐Microbe Interactions, 18, 1130–1139. 16353548 10.1094/MPMI-18-1130

[mpp13155-bib-0041] Khang, C.H. , Berruyer, R. , Giraldo, M.C. , Kankanala, P. , Park, S.Y. , Czymmek, K. et al. (2010) Translocation of *Magnaporthe oryzae* effectors into rice cells and their subsequent cell‐to‐cell movement. The Plant Cell, 22, 1388–1403. 20435900 10.1105/tpc.109.069666 PMC2879738

[mpp13155-bib-0043] Knig, A. , Müller, R. , Mogavero, S. & Hube, B. (2020) Fungal factors involved in host immune evasion, modulation and exploitation during infection. Cellular Microbiology, 23, e13272, . 32978997 10.1111/cmi.13272

[mpp13155-bib-0044] Koeck, M. , Hardham, A.R. & Dodds, P.N. (2011) The role of effectors of biotrophic and hemibiotrophic fungi in infection. Cellular Microbiology, 13, 1849–1857. 21848815 10.1111/j.1462-5822.2011.01665.x PMC3218205

[mpp13155-bib-0045] Kumar, J. , Schafer, P. , Huckelhoven, R. , Langen, G. , Baltruschat, H. , Stein, E. et al. (2002) *Bipolaris sorokiniana*, a cereal pathogen of global concern: cytological and molecular approaches towards better control double dagger. Molecular Plant Pathology, 3, 185–195. 20569326 10.1046/j.1364-3703.2002.00120.x

[mpp13155-bib-0046] Kumar, S. , Kumar, N. , Prajapati, S. & Maurya, S. (2020) Review on spot blotch of wheat: an emerging threat to wheat basket in changing climate. Journal of Pharmacognosy and Phytochemistry, 9, 1985–1997.

[mpp13155-bib-0047] Li, C. , Wang, L. , Lin, Y. , Luo, C. & Yin, W. (2019) Functional identification of Six1‐like effector UvSix1‐1 in *Ustilaginoidea virens* . Acta Phytopathologica Sinica, 49, 27–34.

[mpp13155-bib-0105] Li, H. , Wang, H. , Jing, M. , Zhu J. , Guo B. , Wang Y. et al. (2018) A *Phytophthora* effector recruits a host cytoplasmic transacetylase into nuclear speckles to enhance plant susceptibility. eLife, 7, e40039. 30346270 10.7554/eLife.40039 PMC6249003

[mpp13155-bib-0048] Li, M. , Zheng, P. , Huai, B. , Li, D. , Kang, Z. & Liu, J. (2016) Cloning and functional analysis of PsPL1 from *Puccinia striiformis* f.sp.*tritici* . Journal of Northwest A&F University (Nat. Sci. Ed.), 44, 155–160.

[mpp13155-bib-0049] Li, Q.Y. , Qin, Z. , Jiang, Y.M. , Shen, C.C. , Duan, Z.B. & Niu, J.S. (2014) Screening wheat genotypes for resistance to black point and the effects of diseased kernels on seed germination. Journal of Plant Diseases & Protection, 121, 79–88.

[mpp13155-bib-0050] Li, S. , Miu, Z. & Gao, W. (2011) Challenges, opportunities and obligations in manegement of soilborne plant disease in China. Chinese Journal of Biological Control, 27, 433–440.

[mpp13155-bib-0051] Li, Z. , Yin, Z. , Fan, Y. , Xu, M. , Kang, Z. & Huang, L. (2015) Candidate effector proteins of the necrotrophic apple canker pathogen *Valsa mali* can suppress BAX‐induced PCD. Frontiers in Plant Science, 6, 579. 26284095 10.3389/fpls.2015.00579 PMC4515548

[mpp13155-bib-0052] Liu, H. , Feng, D. , Liu, B. , He, Y. , Wand, H. & Wang, J. (2009) Studies on subcellular localization of MpASR in onion epidermal cells mediated by *Agrobacterium* . Journal of Tropical and Subtropical Botany, 17, 218–222.

[mpp13155-bib-0040] Livak, K.J. & Schmittgen, T.D. (2001) Analysis of relative gene expression data using real‐time quantitative PCR and the 2^−ΔΔCT^ method. Methods, 25, 402–408. 11846609 10.1006/meth.2001.1262

[mpp13155-bib-0053] Luan, F. , Qing, S. & Duan, X. (2011) Study on the pathogenetic fungi of the black point disease of wheat and their characteristics of infestation in Xinjiang. Xinjiang Agricultural Sciences, 48, 2223–2229.

[mpp13155-bib-0054] Lyu, X. , Shen, C. , Fu, Y. , Xie, J. , Jiang, D. , Li, G. et al. (2016) A small secreted virulence‐related protein is essential for the necrotrophic interactions of *Sclerotinia sclerotiorum* with its host plants. PLoS Pathogens, 12, e1005435. 26828434 10.1371/journal.ppat.1005435 PMC4735494

[mpp13155-bib-0055] Mali, K. , Mirajkar, K.K. , Biradar, S. & Patil, S.R. (2017) Role of defence related enzymes and liginin host resistance of durum wheat cultivars infected by *Bipolaris sorokiniana* . International Journal of Agriculture Sciences, 9, 3886–3890.

[mpp13155-bib-0056] Mafurah, J.J. , Ma, H. , Zhang, M. , Xu, J. , He, F. , Ye, T. et al. (2015) A virulence essential CRN effector of *Phytophthora capsici* suppresses host defense and induces cell death in plant nucleus. PLoS One, 10, 1–15. 10.1371/journal.pone.0127965 PMC4444017 26011314

[mpp13155-bib-0057] Malik, N.A.A. , Kumar, I.S. & Nadarajah, K. (2020) Elicitor and receptor molecules: orchestrators of plant defense and immunity. International Journal of Molecular Sciences, 21, 963. 10.3390/ijms21030963 PMC7037962 32024003

[mpp13155-bib-0058] Meenakshi, T. & Singh, S.B. (2013) Role of elicitors in inducing resistance in plants against pathogen infection: a review. ISRN Biochemistry, 2013, 762412. 25969762 10.1155/2013/762412 PMC4393000

[mpp13155-bib-0059] Murray, T.D. , Parry, D.W. & Cattlin, N.D. (1998) A colour handbook of diseases of small grain cereal crops. A Color Handbook of Diseases of Small Grain Cereal Crops, 49(3), 402.

[mpp13155-bib-0060] Naz, R. , Nosheen, A. , Yasmin, H. , Bano, A. & Keyani, R. (2018) Botanical‐chemical formulations enhanced yield and protection against *Bipolaris sorokiniana* in wheat by inducing the expression of pathogenesis‐related proteins. PLoS One, 13, e0196194. 29708983 10.1371/journal.pone.0196194 PMC5927443

[mpp13155-bib-0062] Niu, J. , Liu, R. & Zheng, L. (2007) Expression analysis of wheat PR‐1, PR‐2, PR‐5 activated by Bgt and SA, and powdery mildew resistance. Journal of Triticeae Crops, 27, 1132–1137.

[mpp13155-bib-0063] Park, H.S. & Yu, J.H. (2012) Genetic control of asexual sporulation in filamentous fungi. Current Opinion in Microbiology, 15, 669–677. 23092920 10.1016/j.mib.2012.09.006

[mpp13155-bib-0064] Pathak, G.M. , Gurjar, G.S. & Kadoo, N.Y. (2020) Insights of *Bipolaris sorokiniana* secretome‐an in silico approach. Biologia, 75, 2367–2381.

[mpp13155-bib-0066] Plett, J. , Kemppainen, M. , Kale, S. , Kohler, A. , Legué, V. , Brun, A. et al. (2011) A secreted effector protein of *Laccaria bicolor* is required for symbiosis development. Current Biology, 21, 1197–1203. 21757352 10.1016/j.cub.2011.05.033

[mpp13155-bib-0067] Pramod, P. , Siddanna, S. , Bhardwaj, S.C. , Gangwar, O.P. & Kumar, S. (2019) Rust pathogen effectors: perspectives in resistance breeding. Planta, 72, 23–34. 10.1007/s00425-019-03167-6 30980247

[mpp13155-bib-0068] Rafiqi, M. , Gan, P.H.P. , Ravensdale, M. , Lawrence, G.J. , Ellis, J.G. , Jones, D.A. et al. (2010) Internalization of flax rust avirulence proteins into flax and tobacco cells can occur in the absence of the pathogen. The Plant Cell, 22, 2017–2032. 20525849 10.1105/tpc.109.072983 PMC2910983

[mpp13155-bib-0069] Redkar, A. , Hoser, R. , Schilling, L. , Zechmann, B. , Krzymowska, M. , Walbot, V. et al. (2015) A secreted effector protein of *Ustilago maydis* guides maize leaf cells to form tumors. The Plant Cell, 27, 1332–1351. 25888589 10.1105/tpc.114.131086 PMC4558682

[mpp13155-bib-0070] Rep, M. , Meijer, M. , Houterman, P.M. , Does, H. & Cornelissen, B. (2005) *Fusarium oxysporum* evades I‐3 ‐mediated resistance without altering the matching avirulence gene. Molecular Plant‐Microbe Interactions, 18, 15–23. 15672814 10.1094/MPMI-18-0015

[mpp13155-bib-0071] Rocha, R.O. , Elowsky, C. , Pham, N.T.T. & Wilson, R.A. (2020) Spermine‐mediated tight sealing of the *Magnaporthe oryzae* appressorial pore–rice leaf surface interface. Nature Microbiology, 5, 1472–1480. 10.1038/s41564-020-0786-x 32929190

[mpp13155-bib-0072] Sahu, R. , Sharaff, M. , Pradhan, M. , Sethi, A. , Bandyopadhyay, T. , Mishra, V.K. et al. (2016) Elucidation of defense‐related signaling responses to spot blotch infection in bread wheat (*Triticum aestivum* L.). The Plant Journal, 86, 35–49. 26932764 10.1111/tpj.13149

[mpp13155-bib-0073] Selin, C. , de Kievit, T.R. , Belmonte, M.F. & Fernando, W.G. (2016) Elucidating the role of effectors in plant‐fungal interactions: progress and challenges. Frontiers in Microbiology, 7, 600. 27199930 10.3389/fmicb.2016.00600 PMC4846801

[mpp13155-bib-0074] Sharma, R.C. & Duveiller, E. (2010) Spot blotch continues to cause substantial grain yield reductions under resource‐limited farming conditions. Journal of Phytopathology, 154, 482–488.

[mpp13155-bib-0075] Sharpee, W.C. & Dean, R.A. (2016) Form and function of fungal and oomycete effectors. Fungal Biology Reviews, 30, 62–73.

[mpp13155-bib-0076] Shen, Q. , Liu, Y. & Naqvi, N.I. (2018) Fungal effectors at the crossroads of phytohormone signaling. Current Opinion in Microbiology, 46, 1–6. 29452844 10.1016/j.mib.2018.01.006

[mpp13155-bib-0077] Singh, U.B. , Singh, S. , Malviya, D. , Karthikeyan, N. , Imran, M. , Chaurasia, R. et al. (2019) Integration of anti‐penetrant tricyclazole, signaling molecule salicylic acid and root associated *Pseudomonas fluorescens* enhances suppression of *Bipolaris sorokiniana* in bread wheat (*Triticum aestivum* L.). Journal of Plant Pathology, 101, 943–954.

[mpp13155-bib-0078] Song, P. , Tan, C. , Guo, J. , Qi, T. , Liu, P. & Guo, J. (2016) Spatial and temporal expression pattern of effector protein gene PSTG_23616 in *Puccinia striiformis* f.sp. *tritici* . Acta Agriculturae Boreali‐occidentalis Sinica, 25, 1279–1288.

[mpp13155-bib-0079] Stergiopoulos, I. & de Wit, P.J. (2009) Fungal effector proteins. Annual Review of Phytopathology, 47, 233–263. 10.1146/annurev.phyto.112408.132637 19400631

[mpp13155-bib-0080] Sudhir, N. , Singh, Y.P. , Ramesh, C. , Kumar, M.V. , Kumar, V.N. , Kumar, M.P. et al. (2020) ToxA‐Tsn1 interaction for spot blotch susceptibility in Indian wheat: an example of inverse gene‐for‐gene relationship. Plant Disease, 104, 71–81. 31697221 10.1094/PDIS-05-19-1066-RE

[mpp13155-bib-0081] Verma, S.K. , Chaurasia, S.K. , Pankaj, Y.K. & Kumar, R. (2020) Study on the genetic variability and pathogenicity assessment among isolates of spot blotch causing fungi (*Bipolaris sorokiniana*) in wheat (*Triticum aestivum* L.). Plant Physiology Reports, 25, 255–267.

[mpp13155-bib-0082] Vlot, A.C. , Dempsey, D.A. & Klessig, D.F. (2009) Salicylic acid, a multifaceted hormone to combat disease. Annual Review of Phytopathology, 47, 177–206. 10.1146/annurev.phyto.050908.135202 19400653

[mpp13155-bib-0083] Wang, G. , Wang, C. , Hou, R. , Zhou, X. , Li, G. , Zhang, S. et al. (2012) The AMT1 arginine methyltransferase gene is important for plant infection and normal hyphal growth in *Fusarium graminearum* . PLoS One, 7, e38324. 22693618 10.1371/journal.pone.0038324 PMC3365026

[mpp13155-bib-0084] Wang, L. , Zhang, Y. , Du, Z. , Kang, R. , Chen, L. , Xing, X. et al. (2017) *FpPDE1* function of *Fusarium pseudograminearum* on pathogenesis in wheat. Journal of Integrative Agriculture, 16, 2504–2512.

[mpp13155-bib-0085] Wang, N. , Li, Y. , Chen, W. , Yang, H.Z. , Zhang, P.H. & Wu, Y.F. (2018) Identification of *wheat blue dwarf phytoplasma* effectors targeting plant proliferation and defence responses. Plant Pathology, 67, 603–609.

[mpp13155-bib-0086] Wang, Q. , Han, C. , Ferreira, A.O. , Yu, X. , Ye, W. , Tripathy, S. et al. (2011) Transcriptional programming and functional interactions within the *Phytophthora sojae* RXLR effector repertoire. The Plant Cell, 23, 2064–2086. 21653195 10.1105/tpc.111.086082 PMC3160037

[mpp13155-bib-0087] Wang, R. , Leng, Y. & Zhong, S. (2015) The regulatory gene VosA affects conidiogenesis and is involved in virulence of the fungal cereal pathogen *Cochliobolus sativus* . Fungal Biology, 119, 884–900. 26399184 10.1016/j.funbio.2015.06.009

[mpp13155-bib-0088] Wen, Z. , Tian, H. , Xia, Y. & Jin, K. (2020) MaPmt1, a protein O‐mannosyltransferase, contributes to virulence through governing the appressorium turgor pressure in *Metarhizium acridum* . Fungal Genetics and Biology, 145, 103480. 33130254 10.1016/j.fgb.2020.103480

[mpp13155-bib-0089] Weßling, R. , Epple, P. , Altmann, S. , He, Y. , Yang, L.I. , Henz, S. et al. (2014) Convergent targeting of a common host protein‐network by pathogen effectors from three kingdoms of life. Cell Host & Microbe, 16, 364–375. 25211078 10.1016/j.chom.2014.08.004 PMC4191710

[mpp13155-bib-0090] Wit, P.J.G.M.D. , Mehrabi, R. , Burg, H.A.V.D. & Stergiopoulos, I. (2010) Fungal effector proteins: past, present and future. Molecular Plant Pathology, 10, 735–747. 10.1111/j.1364-3703.2009.00591.x PMC6640362 19849781

[mpp13155-bib-0091] Wu, D. , Yu, Q. , Lu, C. & Hengsdijk, H. (2006) Quantifying production potentials of winter wheat in the North China Plain. European Journal of Agronomy, 24, 226–235.

[mpp13155-bib-0092] Xia, Y. , Ma, Z. , Qiu, M. , Guo, B. & Wang, Y. (2020) N‐glycosylation shields *Phytophthora sojae* apoplastic effector PsXEG1 from a specific host aspartic protease. Proceedings of the National Academy of Sciences of the United States of America, 117, 27685–27693. 33082226 10.1073/pnas.2012149117 PMC7959567

[mpp13155-bib-0093] Xu, F. , Yang, G. , Wang, J. , Song, Y. , Liu, L. , Zhao, K. et al. (2018) Spatial distribution of root and crown rot fungi associated with winter wheat in the North China Plain and its relationship with climate variables. Frontiers in Microbiology, 9, 1054. 29887840 10.3389/fmicb.2018.01054 PMC5981207

[mpp13155-bib-0094] Yan, L. , Wang, X. , Xu, R. , Dongfang, Y. & Li, H. (2012) Root and leaf infection as revealed by autofluorescent reporter protein GFP labeled *Bipolaris sorokiniana* in Wheat. Scientia Agricultura Sinica, 45, 3506–3514.

[mpp13155-bib-0095] Yang, G. , Tang, L. , Gong, Y. , Xie, J. , Fu, Y. , Jiang, D. et al. (2018) A cerato‐platanin protein SsCP1 targets plant PR1 and contributes to virulence of *Sclerotinia sclerotiorum* . New Phytologist, 217, 739–755. 10.1111/nph.14842 29076546

[mpp13155-bib-0096] Yoshino, K. , Irieda, H. , Sugimoto, F. , Yoshioka, H. & Takano, Y. (2012) Cell death of *Nicotiana benthamiana* is induced by secreted protein NIS1 of *Colletotrichum orbiculare* and is suppressed by a homologue of CgDN3. Molecular Plant‐Microbe Interactions, 25, 625–636. 22352720 10.1094/MPMI-12-11-0316

[mpp13155-bib-0097] Zhang, J. , Du, X. , Wang, Q. , Chen, X. , Lv, D. , Xu, K. et al. (2010) Expression of pathogenesis related genes in response to salicylic acid, methyl jasmonate and 1‐aminocyclopropane‐1‐carboxylic acid in *Malus hupehensis* (Pamp.) Rehd. BMC Research Notes, 3, 208. 20659347 10.1186/1756-0500-3-208 PMC3161363

[mpp13155-bib-0098] Zhang, J. , Sun, J. , Duan, A. , Wang, J. , Shen, X. & Liu, X. (2007) Effects of different planting patterns on water use and yield performance of winter wheat in the Huang‐Huai‐Hai plain of China. Agricultural Water Management, 92, 41–47.

[mpp13155-bib-0099] Zhang, L. , Ni, H. , Du, X. , Wang, S. , Ma, X.W. , Nürnberger, T. et al. (2017) The *Verticillium*‐specific protein VdSCP7 localizes to the plant nucleus and modulates immunity to fungal infections. New Phytologist, 215, 368–381. 10.1111/nph.14537 28407259

[mpp13155-bib-0100] Zhang, S. & Xu, J.R. (2014) Effectors and effector delivery in *Magnaporthe oryzae* . PLoS Path, 10, e1003826, . 10.1371/journal.ppat.1003826 PMC3879361 24391496

[mpp13155-bib-0101] Zhang, T.Y. , Wang, H.L. & Xu, F.L. (1990) Effects of grain black point disease of wheat and the pathogenic fungi. Acta Phytophylacica Sinica, 17, 313–316.

[mpp13155-bib-0102] Zhang, N. , Yang, J. , Fang, A. , Wang, J. , Li, D. , Li, Y. et al. (2020) The essential effector SCRE1 in *Ustilaginoidea virens* suppresses rice immunity via a small peptide region. Molecular Plant Pathology, 21, 445–459. 32087618 10.1111/mpp.12894 PMC7060142

[mpp13155-bib-0103] Zhang, Y. , Wang, L. , Liang, S. , Zhang, P. , Kang, R. , Zhang, M. et al. (2020) FpDep1, a component of Rpd3L histone deacetylase complex, is important for vegetative development, ROS accumulation, and pathogenesis in *Fusarium pseudograminearum* . Fungal Genetics and Biology, 135, 103299. 31706014 10.1016/j.fgb.2019.103299

[mpp13155-bib-0104] Zhao, Y. , Su, H. , Zhou, J. , Feng, H. , Zhang, K.Q. & Yang, J. (2015) The APSES family proteins in fungi: Characterizations, evolution and functions. Fungal Genetics & Biology, 81, 271–280. 25534868 10.1016/j.fgb.2014.12.003

