# Supplementary figures and images for: A novel effector, CsSp1, from Bipolaris sorokiniana, is essential for colonization in wheat and is also involved in triggering host immunity
Source: Mol Plant Pathol. 2021 Nov 6;23(2):218–36. doi: 10.1111/mpp.13155 (PMC8743017; doi:10.1111/mpp.13155)

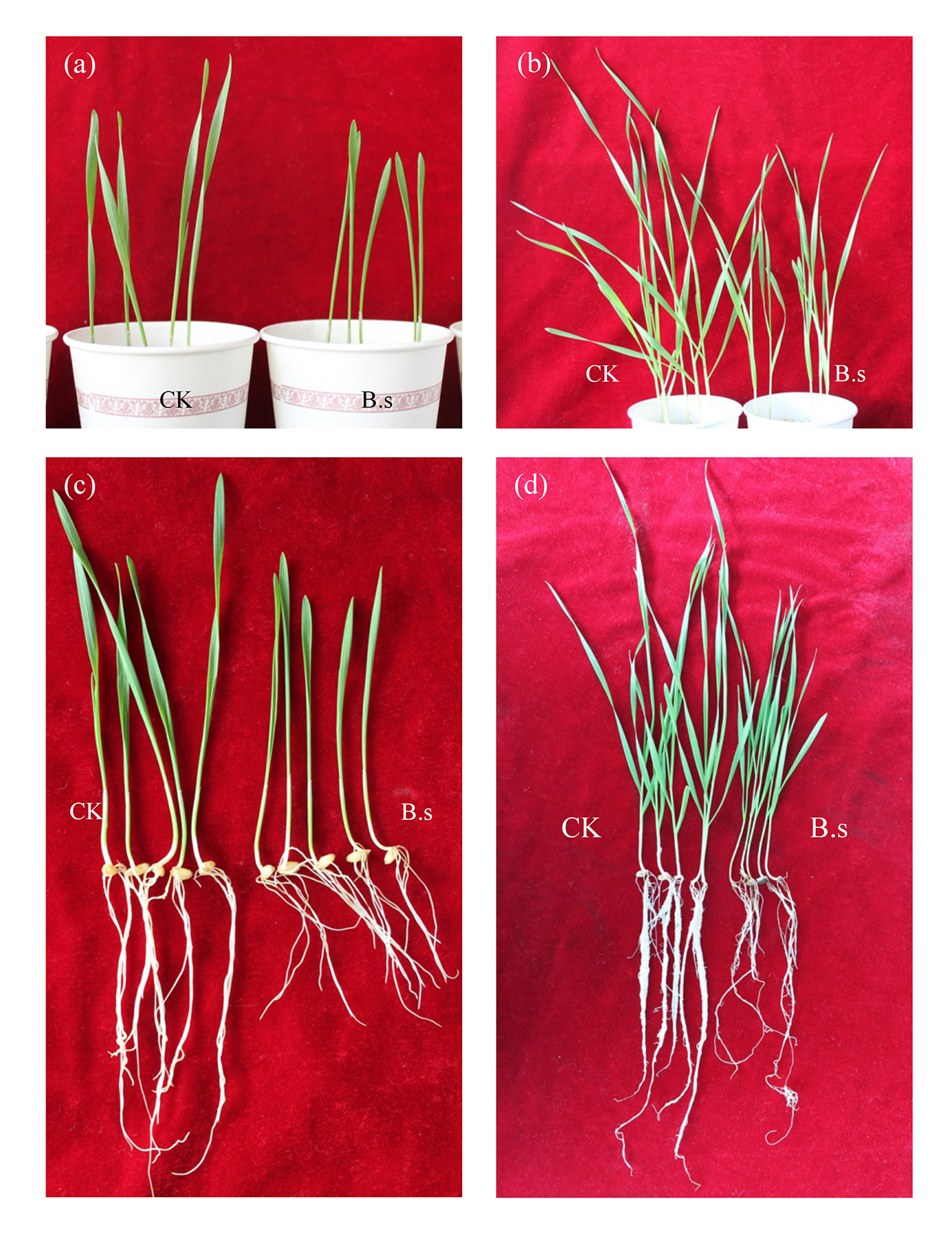

Supplement: Supplementary file 1 — FIGURE S1 Root rot of wheat caused by Bipolaris sorokiniana (the samples of which were used for transcriptome sequencing). (a, b) Seedlings and roots at 5 days and (c, d) 15 days after inoculation. Mock, wheat without inoculation; B.s, inoculation with B. sorokiniana [file MPP-23-218-s005.tif]

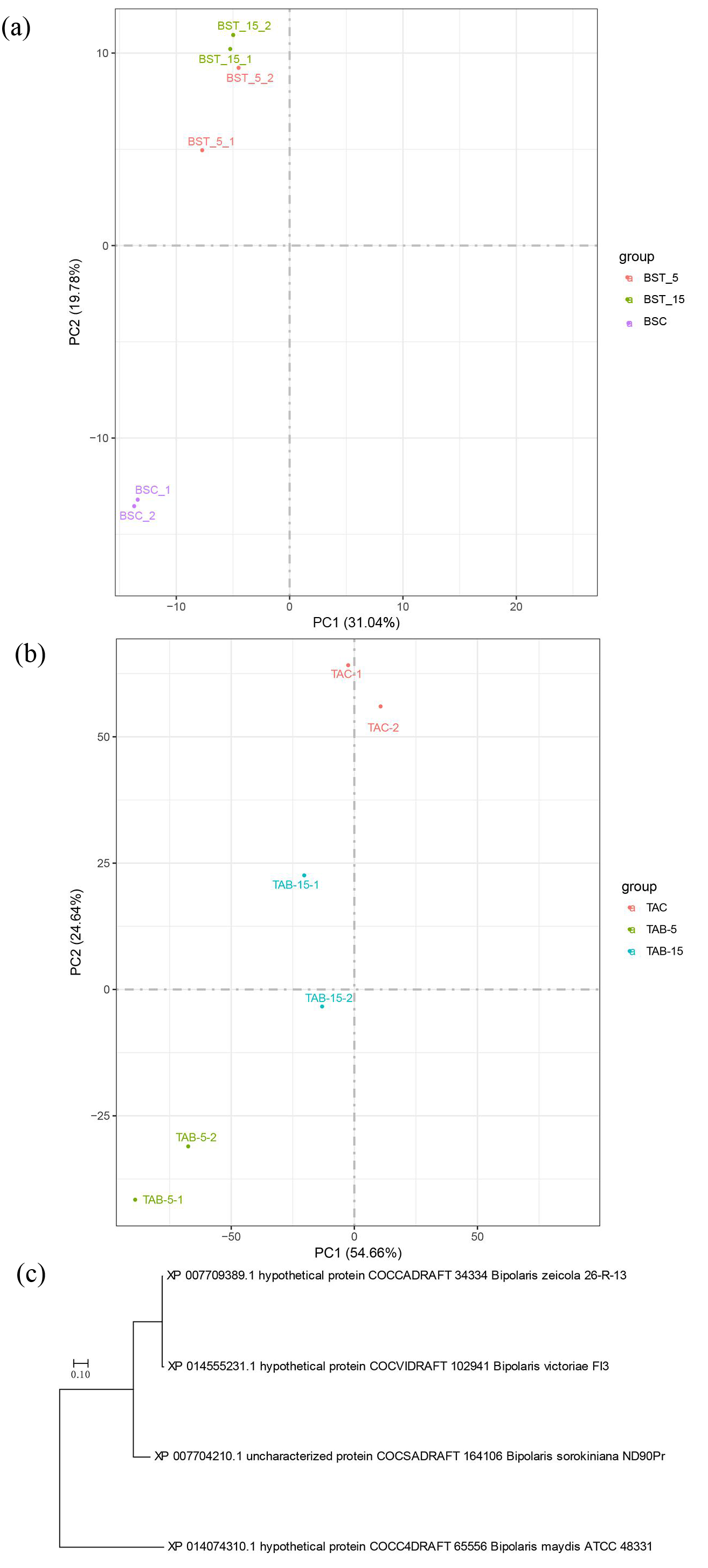

Supplement: Supplementary file 2 — FIGURE S2 (a) Principal component analysis (PCA) of RNA‐Seq data of Bipolaris sorokiniana. (b) Principal component analysis (PCA) of RNA‐Seq data of Triticum aestivum. (c) Phylogenetic tree of orthologues of CsSp1 generated through MEGA 7. Bar, 0.1 [file MPP-23-218-s003.tif]

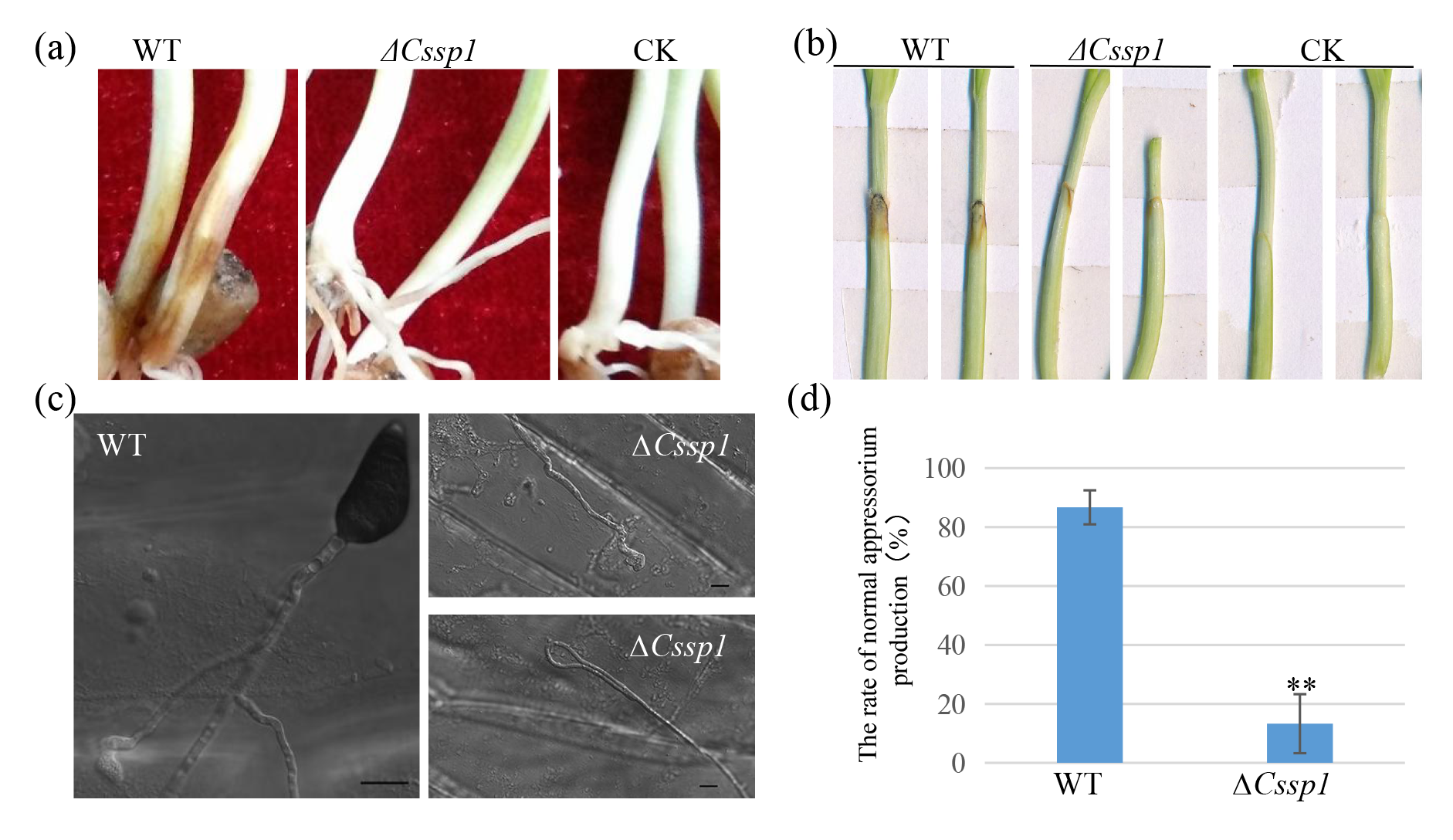

Supplement: Supplementary file 3 — FIGURE S3 (a) Pathogenicity test with millet inoculum of ∆Cssp1 and the wild‐type (WT) strain applied to the soil. (b) Three days after inoculation of wheat coleoptiles with agar blocks of fungal cultures. (c) Spore germination and appressorium formation of ∆Cssp1 on onion epidermal cells compared with WT cells. (d) The rate of normal appressorium production [file MPP-23-218-s002.tif]

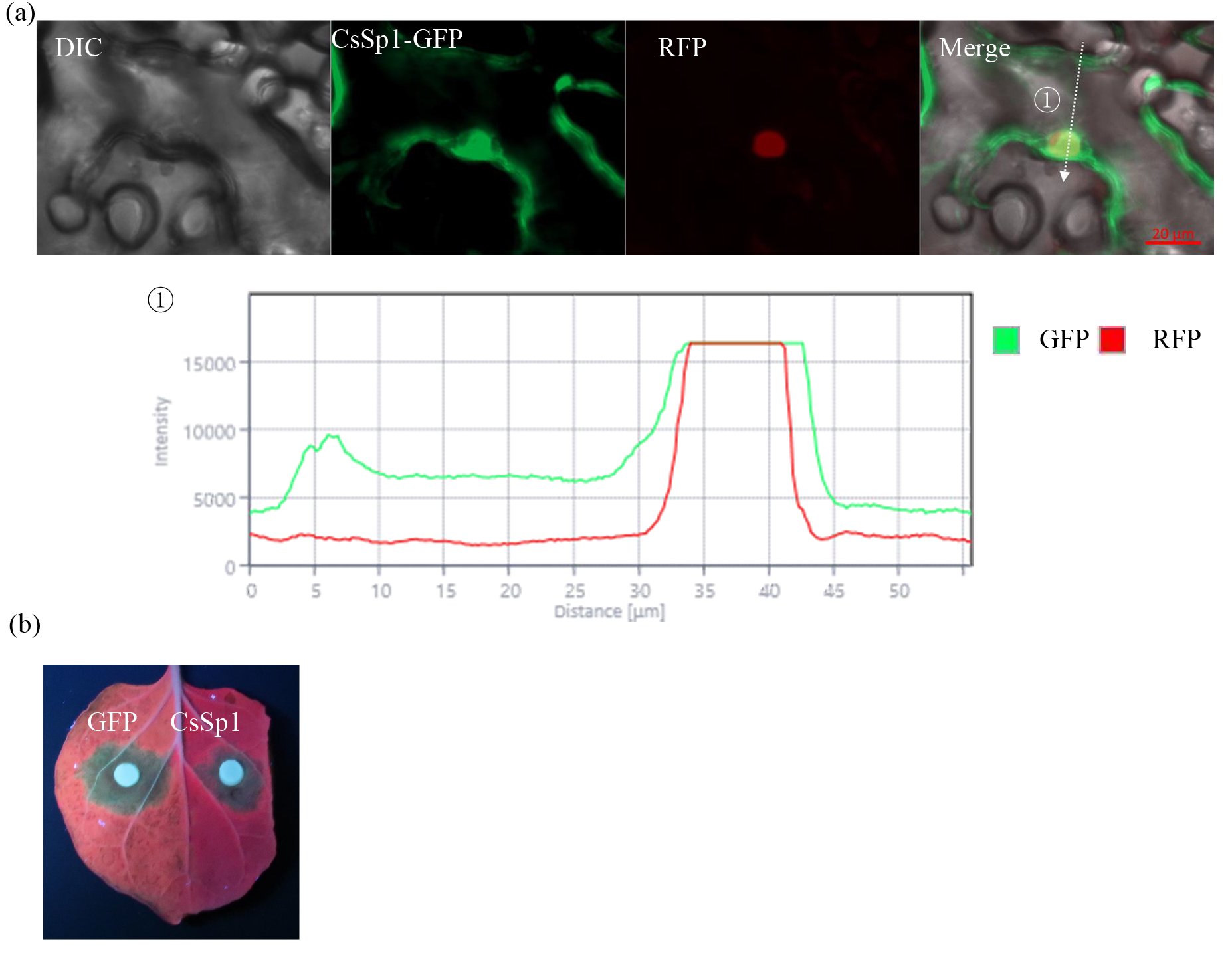

Supplement: Supplementary file 4 — FIGURE S4 (a) Subcellular localization of CsSp1‐GFP in H2B‐red transgenic Nicotiana benthamiana leaves. Bar, 20 μm. ① The signal peaks of CsSP1 green and H2B red obviously merged together. The white arrow shows the obvious co‐location site. (b) Independent replicates of those shown in Figure 5g [file MPP-23-218-s004.tif]

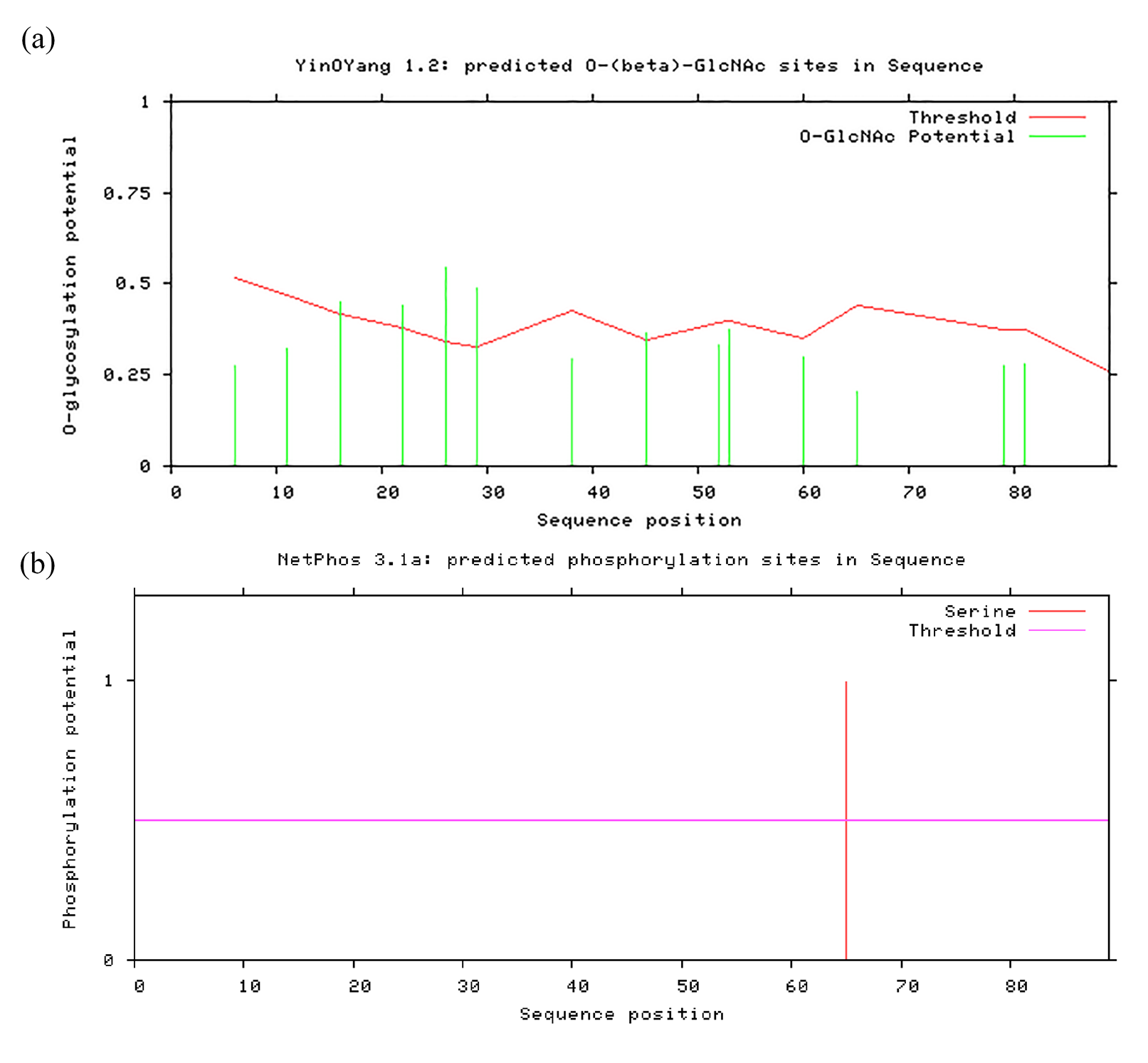

Supplement: Supplementary file 5 — FIGURE S5 (a) Prediction of CsSp1 O‐glycosylation sites via YinOYang 1.2. (b) Prediction of CsSp1 phosphorylation sites via NetPhos 3.1a. The threshold was 0.6 [file MPP-23-218-s001.tif]
